# ACSS2‐Mediated Histone H4 Lysine 12 Crotonylation (H4K12cr) Alleviates Colitis via Enhancing Transcription of CLDN7

**DOI:** 10.1002/advs.202500461

**Published:** 2025-07-12

**Authors:** Ming Yuan, Shaopeng Chen, Zhensen Lin, Runfeng Yu, Kang Chao, Shubiao Ye, Qing Li, Haoxian Ke, Chi Zhang, Junfeng Huang, Guanzhan Liang, Tuo Hu, Xiang Gao, Ping Lan, Xianrui Wu

**Affiliations:** ^1^ Department of General Surgery (Colorectal Surgery) The Sixth Affiliated Hospital Sun Yat‐sen University Guangzhou Guangdong 510655 China; ^2^ Guangdong Provincial Key Laboratory of Colorectal and Pelvic Floor Diseases The Sixth Affiliated Hospital Sun Yat‐sen University Guangzhou Guangdong 510655 China; ^3^ Biomedical Innovation Center The Sixth Affiliated Hospital Sun Yat‐sen University Guangzhou Guangdong 510655 China; ^4^ Department of Gastroenterology The Sixth Affiliated Hospital Sun Yat‐sen University Guangzhou Guangdong 510655 China; ^5^ Department of Gastrointestinal Surgery Sun Yat‐sen Memorial Hospital Sun Yat‐sen University Guangzhou Guangdong 510288 China; ^6^ State Key Laboratory of Oncology in South China Guangzhou Guangdong 510288 China

**Keywords:** ACSS2, inflammatory bowel disease, intestinal barrier, histone lysine crotonylation

## Abstract

Histone lysine crotonylation (Kcr), a highly conserved posttranslational modification, plays critical roles in various biological processes. Nevertheless, the dynamic alterations and functions of histone Kcr in inflammatory bowel disease (IBD) remain poorly explored. Herein, a notable decrease of both Pan‐Kcr and ACSS2 (acyl‐CoA synthetase short‐chain family member 2), the key enzyme for crotonyl‐CoA generation, is revealed in inflamed intestinal epithelial cells. Genetic or pharmacological inhibition of ACSS2 dramatically impairs mouse intestinal barrier integrity and exacerbates colitis. Mechanistically, ACSS2‐mediated histone H4 lysine 12 crotonylation (H4K12cr) upregulates CLDN7 expression to fortify intestinal epithelial barrier, which can be augmented by crotonate supplementation. Furthermore, tumor necrosis factor‐α (TNF‐α) is revealed to enhance the m6A modification of *ACSS2* mRNA, consequently destabilizing and downregulating *ACSS2*. Combinational therapy involving anti‐TNF‐α and crotonate can significantly ameliorate colitis. Overall, ACSS2‐mediated H4K12cr emerges as a pivotal modulator governing intestinal barrier function during IBD progression.

## Graphical abstract

Histone lysine crotonylation (Kcr) is a highly conserved posttranslational modification implicated with crucial biological processes. This study reveals that ACSS2‐mediated H4K12cr upregulated *CLDN7* transcription to strengthen intestinal epithelial barrier. TNF‐α enhanced the m6A modification of *ACSS2* mRNA, leading to the downregulation of ACSS2‐mediated H4K12cr. This study emphasizes the importance of ACSS2‐H4K12cr‐CLDN7 axis in intestinal barrier function and IBD progression.

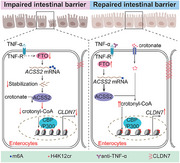

## 1 Introduction

Inflammatory bowel disease (IBD), including Crohn's disease (CD) and ulcerative colitis (UC), presents as chronic and relapsing inflammatory disorder in intestine. IBD has become a serious clinical burden, with a prevalence exceeding 0.3% worldwide.^[^ ^1^, ^2^ ^]^ One typical hallmark of IBD is intestinal barrier destruction, which leads to the translocation of pathogens or endotoxins and subsequent dysregulated immune responses.^[^ ^2^, ^3^ ^]^ Within the intestinal epithelial cells, tight junction molecules play an essential role in mechanical barrier maintenance.^[^ ^4^, ^5^, ^6^ ^]^ However, these tight junction proteins are dramatically decreased in inflamed intestinal epithelium.^[^ ^7^, ^8^, ^9^ ^]^ Recently, the concept of mucosal healing has been established as a vital therapeutic goal in clinical management for IBD, which can reduce adverse outcomes and enhance clinical remission rates.^[^ ^3^, ^10^, ^11^ ^]^ Therefore, the restoration of intestinal epithelial barrier function is essential for IBD management.

Protein posttranslational modifications (PTMs) are crucial for regulating various biological processes and are implicated in disorders, including inflammatory diseases like IBD.^[^ ^12^, ^13^, ^14^ ^]^ Aberrant PTMs in intestinal epithelium have been proven to contribute to IBD progression.^[^ ^15^, ^16^ ^]^ Lysine crotonylation (Kcr) is a highly conserved PTM, utilizing crotonyl‐CoA as its substrate, which could be produced from crotonate conversion, fatty acid oxidation, and lysine or tryptophan catabolism.^[^ ^17^, ^18^ ^]^ The level of protein Kcr is regulated by crotonyl‐transferases (writers), decrotonylases (erasers), and crotonyl‐CoA concentration.^[^ ^19^, ^20^, ^21^ ^]^ In the mammalian genome, histone Kcr is particularly enriched at transcriptional start sites (TSS) and enhancer regions, exerting an important effect on gene transcription.^[^ ^21^, ^22^, ^23^, ^24^ ^]^ Research has revealed the critical roles of histone Kcr in various functions such as embryo development,^[^ ^17^, ^25^ ^]^ cancer progression,^[^ ^22^, ^26^ ^]^ chronic kidney disease,^[^ ^27^ ^]^ and so on. However, the specific functional role of histone Kcr in IBD remains poorly explored.

In this work, we elaborated the barrier‐protective role of ACSS2, which functioned as a vital crotonyl‐CoA producer. ACSS2 and Pan‐Kcr level were significantly decreased in inflamed intestinal epithelium of IBD patients. Genetic or pharmacological inhibition of ACSS2 remarkably impaired intestinal barrier integrity and worsened colitis. Mechanistically, ACSS2 enhanced *CLDN7* transcription via histone H4 lysine 12 crotonylation (H4K12cr) modification, which was further strengthened by crotonate supplementation, a precursor for crotonyl‐CoA. Moreover, we identified that TNF‐α destabilized *ACSS2* mRNA by enhancing its m6A modification level, thereby aggravating colitis. More importantly, combining anti‐TNF‐α therapy with crotonate supplementation could effectively alleviate colitis. Overall, this study positions ACSS2‐mediated crotonylation as an important protective factor in IBD pathogenesis, providing valuable insights for clinical management.

## 2 Results

### 2.1 Pan‐Kcr and ACSS2 are Downregulated in Intestinal Epithelium of IBD Patients and Murine Colitis Models

**Figure 1:**
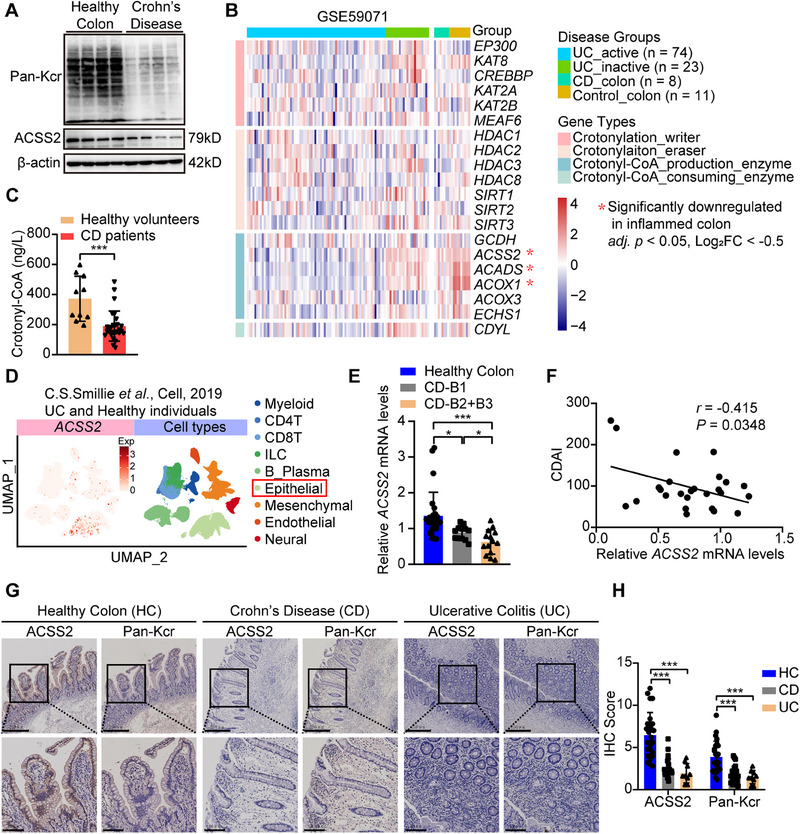
Reduced Pan‐Kcr and ACSS2 expression in intestinal epithelium of IBD patients. A) Immunoblotting of Pan‐Kcr and ACSS2 in colon tissues from healthy controls and CD patients. B) Heatmap of gene expression profiling involved in crotonylation from GSE59071 dataset. Red stars indicate significantly downregulated genes in inflamed colon tissues. C) Comparison of crotonyl‐CoA levels in plasma samples from healthy volunteers (*n* = 10) and CD patients (*n* = 28). D) UMAP visualization of *ACSS2* expression levels across different cell types, analyzed by scIBD platform. E) Comparative analysis of *ACSS2* mRNA expression in colon tissues between healthy control (*n* = 26) and CD patients with clinical stage B1 (*n* = 12) and B2+B3 (*n* = 14). F) Correlation analysis of *ACSS2* mRNA levels in colon tissues and CDAI from CD patients (*n* = 26). G) Representative IHC images of ACSS2 and Pan‐Kcr in colon tissues from healthy control, CD patients, and UC patients. Scale bar = 250 µm (upper) and 50 µm (lower). H) Comparative analysis of ACSS2 and Pan‐Kcr levels (IHC score) in colon tissues among healthy control (*n* = 25), CD patients (*n* = 24), and UC patients (*n* = 9). The mRNA expression profiling of GSE59071 dataset is re‐analyzed by DESeq2 package (version 1.16.1) in R software (version 4.1.2). Values are mean ± SD. *p < 0.05, **p < 0.01, ***p < 0.001, determined by two‐tailed Student's *t*‐test C), one‐way ANOVA with Bonferroni's post hoc test E, H), and Pearson's correlation with two‐tailed test F).

To elucidate the role of protein crotonylation in IBD, the abundance of Pan‐Kcr was primarily assessed, revealing a notable reduction of Pan‐Kcr in inflamed intestinal tissues of both IBD patients and mouse colitis models (**Figure** **1**A; Figure S1A–C, Supporting Information). Given that Kcr homeostasis is coordinately regulated by three key elements: crotonyl‐transferases (writers), decrotonylases (erasers), and substrate availability (crotonyl‐CoA), we conducted transcriptomic analysis of these regulatory components in a public IBD cohort (GSE59071).^[^ ^28^ ^]^ Significant reduction of several enzymes for crotonyl‐CoA synthesis was identified in IBD patients, among which *ACSS2* was downregulated in both human and murine inflamed intestinal tissues (Figure 1B; Figure S1D, Supporting Information). ACSS2 catalyzes crotonyl‐CoA production, thereby facilitating protein crotonylation.^[^ ^21^, ^27^ ^]^ As a source of intracellular acyl‐CoA pools,^[^ ^29^, ^30^, ^31^ ^]^ we detected plasma crotonyl‐CoA levels and observed a moderate decrease in CD patients (Figure 1C). The single‐cell transcriptomics data derived from UC patients and healthy individuals^[^ ^32^ ^]^ showed that *ACSS2* was expressed in intestinal epithelial cells predominantly (Figure 1D) and its expression was decreased in inflamed intestinal epithelial cells (Figure S1E, Supporting Information). Furthermore, *ACSS2* expression was negatively correlated with the progression of CD (Figure 1E,F). Immunohistochemistry results unveiled a remarkable reduction of ACSS2 and Pan‐Kcr protein levels in IBD patients (Figure 1G,H), which was negatively associated with disease severity (Figure S1F–I, Supporting Information). These results indicate that downregulation of ACSS2 may play an important role in IBD pathogenesis.

### 2.2 ACSS2 Inhibition Impairs Intestinal Barrier Function and Aggravates Colitis

**Figure 2:**
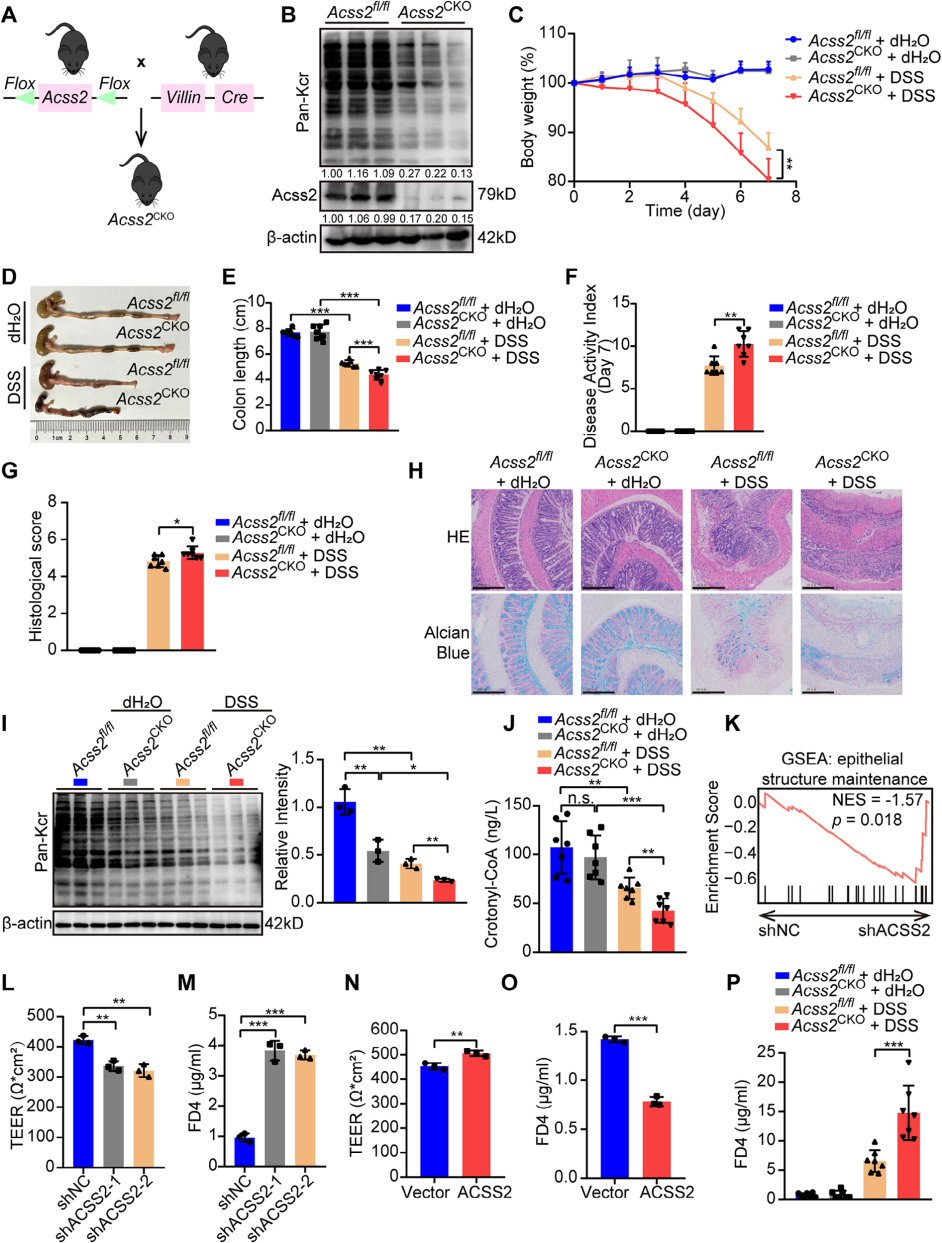
ACSS2 inhibition impairs intestinal barrier function and aggravates colitis. A) The generation strategy for *Acss2* ^CKO^ mice. B) Immunoblotting results for Pan‐Kcr and Acss2 in intestinal tissues from control and *Acss2* ^CKO^ mice. Relative band intensities are annotated below respective lanes. C‐J) ACSS2 knockout in intestinal epithelium aggravates colitis progression (*n* = 7 for each group). C) The mice body weight curves from indicated experimental groups. D, E) Representative colon images and colon length statistics from each group. F) The DAI statistics for mice from indicated groups. G,H) Histological score and representative HE/Alcian blue staining images of colon tissues from indicated groups. Scale bar = 100 µm. I) Immunoblotting results and statistical analysis for Pan‐Kcr protein level in intestinal tissues from indicated groups. J) The plasma crotonyl‐CoA concentration in mice from indicated experimental groups. K) GSEA analysis of differentially expressed genes from control and ACSS2‐knockdown NCM460 cells. L–O) The pivotal role of ACSS2 in barrier function maintenance. The results of TEER and FD4 measurement in monolayers of Caco2 cells with either L,M) ACSS2 knockdown or N, O) ACSS2 overexpression. P) The plasma FD4 level in *Acss2* ^CKO^ and *Acss2^fl/fl^* mice (*n* = 7). Values are mean ± SD. n.s. (not significant, *p* > 0.05), *p < 0.05, **p < 0.01, ***p < 0.001, determined by repeated measures ANOVA with Bonferroni's post hoc test C), one‐way ANOVA with Bonferroni's post hoc test E, I, J, L, M), two‐tailed Student's *t*‐test F, G, N‐P).

To explore whether ACSS2 is involved in IBD progression, *Acss2* intestinal epithelium conditional knockout mice (*Vil1‐cre*, *Acss2^fl/fl^*; *Acss2* ^CKO^) and control mice (*Acss2^fl/fl^*) were generated and confirmed by genotyping (Figure S2A,B, Supporting Information) and immunoblotting (**Figure** **2**A,B). Following induction of colitis, *Acss2* ^CKO^ mice exhibited a pronounced exacerbation of colitis symptoms, characterized by significant weight loss (Figure 2C), colon length shortening (Figure 2D,E), disease activity index (DAI, Figure 2F), and histological score elevation (Figure 2G,H). Additionally, a substantial reduction of pan‐Kcr modification level in colon tissues and plasma crotonyl‐CoA concentration was observed in *Acss2* ^CKO^ mice with colitis (Figure 2I,J).

Moreover, by comparing the microbiota composition of *Acss2^fl/fl^* mice and *Acss2* ^CKO^ mice under homeostatic or colitis conditions, we revealed that under the colitis condition, the Shannon index of the intestinal microbiota decreased significantly in both mice (Figure S2C, Supporting Information), indicating the diversity of intestinal microbiota decreased in colitis mice. Principal coordinates analysis (PCoA) showed distinct bacterial composition among the four groups (Figure S2D, Supporting Information). Analysis of the microbiota composition at the phylum level suggested that under colitis conditions, the *Bacillota* was moderately reduced while the *Bacteroidota* was significantly increased, which was more obvious in the *Acss2* ^CKO^ mice (Figure S2E, Supporting Information). These results collectively suggested that intestinal microbiota might be a contributing factor to the aggravated colitis phenotype observed in *Acss2* ^CKO^ mice.

To reveal the mechanism of how ACSS2 directly suppressed colitis progression, we performed RNA‐seq from control and ACSS2 knockdown NCM460 cells (Figure S3A, Supporting Information). GSEA demonstrated that ACSS2 knockdown negatively correlated with epithelial structure maintenance (Figure 2K). As the intestinal barrier is vital for IBD pathogenesis, we proposed that downregulation of ACSS2 might promote IBD progression by impairing the intestinal barrier. To evaluate the effect of ACSS2 on intestinal epithelial barrier function in vitro, Caco2 cells with either stable knockdown or overexpression of ACSS2 were constructed (Figure S3A,B, Supporting Information) and subjected for TEER and FD4 permeability assays. Notably, ACSS2 knockdown remarkably impaired barrier function, manifested as TEER reduction and FD4 permeability increase (Figure 2L,M), whereas ACSS2 overexpression enhanced barrier function (Figure 2N,O). ACSS2 enzymatic inhibitor, VY‐3‐135,^[^ ^33^ ^]^ could downregulate intracellular crotonyl‐CoA level (Figure S3C, Supporting Information), accompanied with diminished TEER and increased FD4 permeability (Figure S3D,E, Supporting Information). Moreover, the intestinal permeability was obviously impaired in *Acss2* ^CKO^ mice, as demonstrated by FD4 gavage (Figure 2P). In all, these results suggest the crucial role of ACSS2 in colitis progression through maintaining intestinal epithelial barrier integrity.

### 2.3 ACSS2 Enhances Intestinal Epithelial Barrier Function via Upregulating CLDN7 Expression

**Figure 3:**
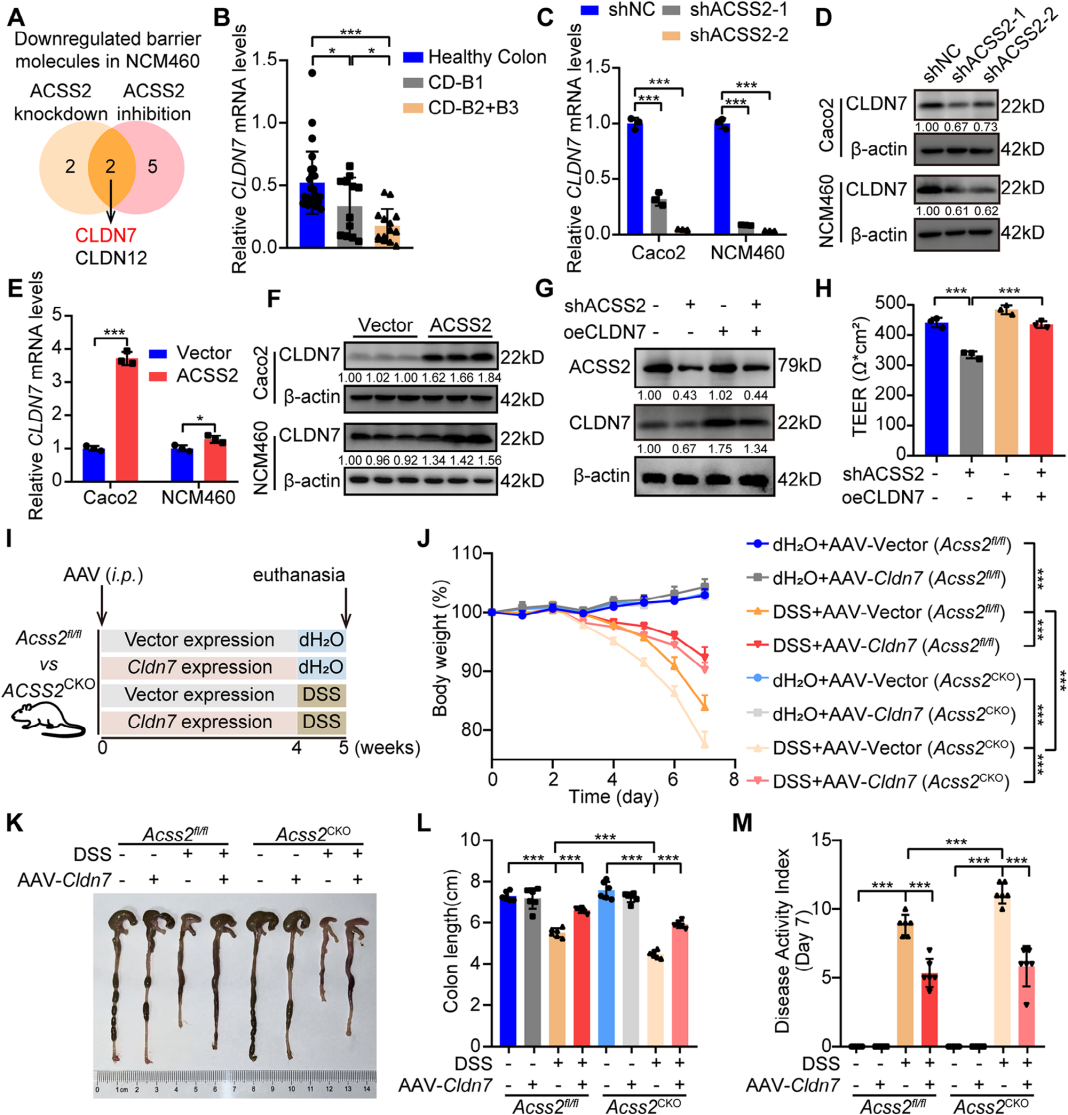
ACSS2 upregulates CLDN7 expression to enhance intestinal epithelial barrier function. A) The screening workflow and Venn diagram for candidate tight junction proteins regulated by ACSS2. B) Comparative analysis of *CLDN7* mRNA expression in colon tissues between healthy control (*n* = 26) and CD patients with clinical stage B1 (*n* = 12) or B2 + B3 (*n* = 14). C,D) CLDN7 mRNA and protein expression levels in control and ACSS2‐knockdown Caco2 and NCM460 cell lines. E, F) CLDN7 mRNA and protein expression levels in control and ACSS2‐overexpression Caco2 and NCM460 cell lines. G) Immunoblotting results for CLDN7 and ACSS2 in indicated Caco2 cells. H) The results of TEER in monolayers of Caco2 cells from above indicated groups. I) The administration strategy of AAV in indicated groups. J–M) CLDN7 overexpression in intestinal epithelium rescued barrier function impairment and colitis progression (*n* = 6 for each group). J) The mice body weight curves from indicated experimental groups. K, L) Representative colon images and colon length statistics from each group. M) The DAI statistics for mice from each group. Relative band intensities are annotated below respective lanes (D, F, G). Values are presented as mean ± SD. *p < 0.05, ***p < 0.001, determined by one‐way ANOVA with Bonferroni's post hoc test B, C, H, L, M), two‐tailed Student's *t*‐test E), and repeated measures ANOVA with Bonferroni's post hoc test J).

Considering the critical function of tight junction in barrier integrity maintenance, we evaluated the mRNA expression levels of tight junction‐associated genes in NCM460 cells with ACSS2 knockdown or inhibition. Our results revealed that ACSS2 knockdown led to the downregulation of four tight junction molecules, while ACSS2 inhibition resulted in the reduction of seven tight junction molecules. By analyzing these downregulated genes, we identified Claudin 7 (CLDN7), crucial for intestinal stem cell homeostasis^[^ ^34^, ^35^ ^]^ and epithelial integrity,^[^ ^7^, ^36^ ^]^ exhibited the most significant downregulation in NCM460 cells with both ACSS2 knockdown and inhibition (**Figure** **3**A). Moreover, a remarkable reduction of *CLDN7* mRNA level was identified in our CD patient cohort, which was negatively correlated with CD progression (Figure 3B). The CLDN7 expression levels were dramatically decreased upon ACSS2 knockdown or VY‐3‐135 treatment (Figure 3C,D; Figure S3F,G, Supporting Information). In contrast, ACSS2 overexpression upregulated CLDN7 expression (Figure 3E,F).

To further confirm whether ACSS2 contributes to intestinal barrier function by regulating CLDN7, ACSS2‐knockdown Caco2 cells were rescued by overexpressing CLDN7 (Figure 3G) for TEER and FD4 evaluation. Remarkably, the impaired barrier function was restored following CLDN7 overexpression, indicating that the barrier‐protective effect of ACSS2 inhibition was dependent on CLDN7 (Figure 3H; Figure S3H, Supporting Information). Furthermore, adeno‐associated virus 9 (AAV9) carrying *Villin*‐promoter for *CLDN7* overexpression specifically in intestinal epithelium was generated and administrated for *Acss2^fl/fl^* mice and *Acss2* ^CKO^ mice, as validated by immunohistochemistry (Figure 3I; Figure S3I, Supporting Information). Intestinal epithelium‐conditional overexpression of CLDN7 effectively ameliorated weight loss (Figure 3J), colon shortening (Figure 3K,L), disease activity (Figure 3M), intestinal barrier function (Figure S3J, Supporting Information), and histological damage (Figure S3K,L, Supporting Information). These results illustrate that ACSS2 enhances intestinal barrier function by upregulating CLDN7 expression.

### 2.4 ACSS2 Upregulates CLDN7 Expression by Enhancing H4K12 Crotonylation

**Figure 4:**
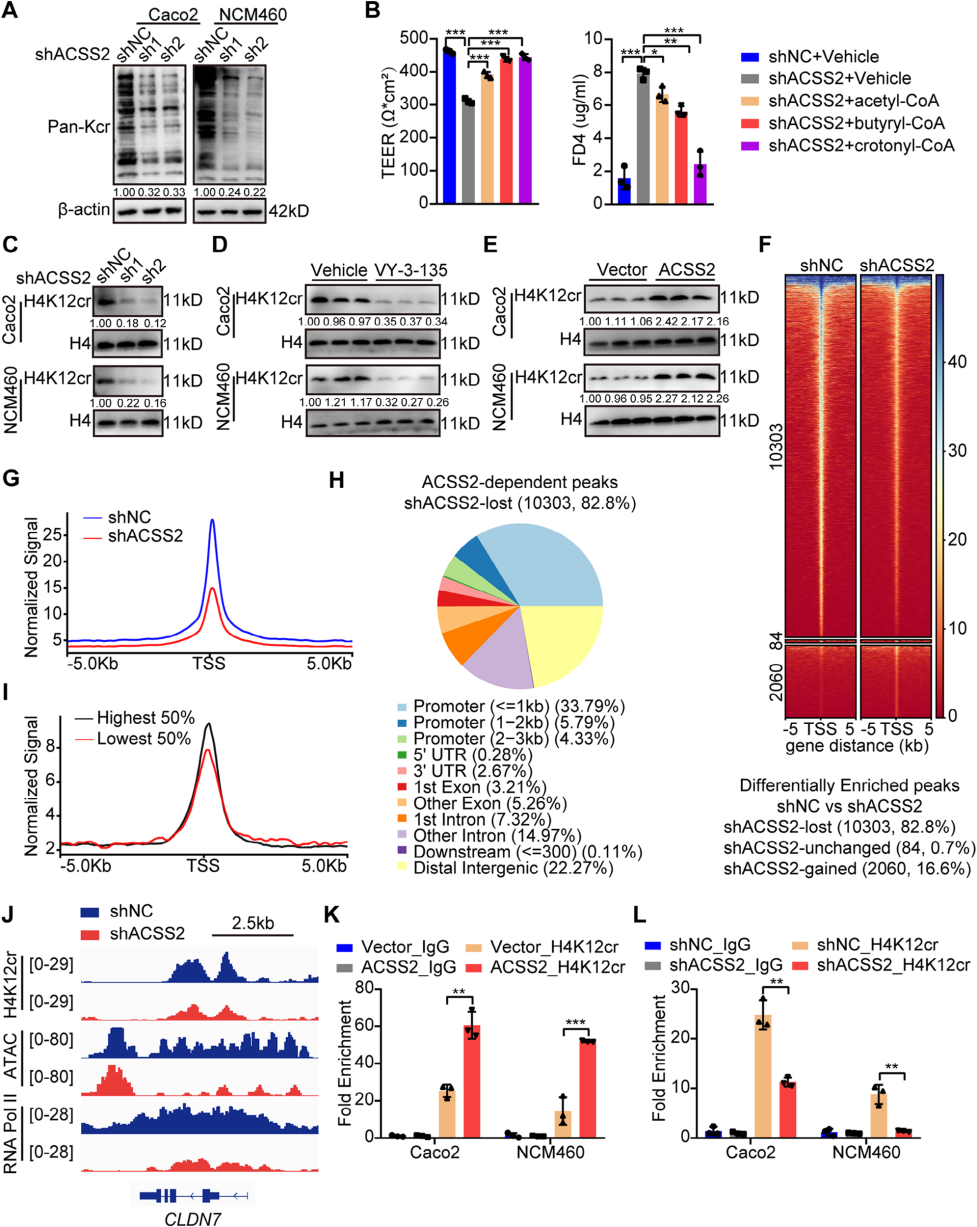
CLDN7 expression is regulated by ACSS2‐mediated H4K12cr. A) Immunoblotting for Pan‐Kcr level in control and ACSS2‐knockdown Caco2 and NCM460 cells. B) TEER and FD4 level in monolayers of Caco2 cells treated with indicated acyl‐CoA (200 µm). Caco2 cells were treated with indicated acyl‐CoA every other day until the successful establishment of the cell barrier model. C–E) Immunoblotting for H4K12cr level in control and C) ACSS2‐knockdown, D) VY‐3‐135 (1 µm) treated or E) ACSS2‐overexpressed cells. F) The heatmap of H4K12cr ChIP‐seq signals sorted by differential H4K12cr peaks in control and ACSS2‐knockdown NCM460 cells. G) The H4K12cr signal profiles around TSS regions in NCM460 cells with ACSS2 knockdown. H) The distribution of ACSS2‐knockdown mediated H4K12cr peaks loss. I) H4K12cr was positively correlated with mRNA expression levels. The ChIP signal intensity (read count per million mapped reads) was shown for genes with differential expressed genes (the top 50% and the bottom 50% of RNA‐seq counts). J) IGV track visulization for *CLDN7* based on indicated ChIP‐seq and ATAC‐seq analysis results. K, L) ChIP‐qPCR analysis results of H4K12cr on CLDN7 in control and ACSS2‐knockdown Caco2 and NCM460 cells. Relative band intensities are annotated below respective lanes (A, C–E). Values are mean ± SD. *p < 0.05, **p < 0.01, ***p < 0.001, determined by one‐way ANOVA with Bonferroni's post hoc test B) and two‐tailed Student's *t*‐test K, L).

Next, we investigated the potential mechanism of how ACSS2 upregulated *CLDN7* expression. Given histone crotonylation plays a vital role in gene transcription, we supposed ACSS2 might regulate CLDN7 expression through modulating histone Kcr. Considering that ACSS2 is also known to regulate lysine acetylation (Kac)^[^ ^37^, ^38^ ^]^ and lysine butyrylation (Kbu),^[^ ^30^ ^]^ control and ACSS2‐knockdown cells were processed to evaluate Pan‐Kcr, Pan‐Kac, and Pan‐Kbu levels. ACSS2 knockdown led to a more pronounced reduction in Pan‐Kcr level compared to Pan‐Kac or Pan‐Kbu levels (**Figure** **4**A; Figure S4A,B, Supporting Information). Moreover, a substantial decrease of crotonyl‐CoA level was observed in ACSS2‐knockdown cells, but less pronounced changes in acetyl‐CoA and butyryl‐CoA level (Figure S4C–E, Supporting Information), while a remarkable increase of crotonyl‐CoA level was observed in ACSS2‐overexpressed cells, but less pronounced changes in acetyl‐CoA and butyryl‐CoA level (Figure S4F–H, Supporting Information). By supplementing acyl‐CoA into Caco2 monolayer cells, we found that crotonyl‐CoA could enhance barrier function and *CLDN7* expression to a greater extent than acetyl‐CoA or butyryl‐CoA (Figure 4B; Figure S4I, Supporting Information), indicating that ACSS2‐knockdown mediated barrier function impairment was more dependent on crotonylation.

Mass spectrometry proteomics analysis indicated Histone H4 was the most abundant histone modified by crotonylation in intestinal epithelial cells (Figure S4J, Supporting Information). Additionally, a panel of acylation antibodies targeting different lysine positions on Histone H4 showed that H4K12 crotonylation (H4K12cr) decreased most significantly after ACSS2 knockdown, with little effects on other H4 lysine crotonylation, H4K12ac, and H4K12bu modification (Figure 4C; Figure S4K, Supporting Information). ACSS2 knockdown also reduced histone Kcr levels at other histone lysine residues, though to a lesser extent (Figure S4K, Supporting Information). Moreover, ACSS2 inhibitor treatment could downregulate H4K12cr level, while ACSS2 overexpression possessed the opposite effect (Figure 4D,E), suggesting that H4K12cr might be involved in ACSS2‐regulated gene expression. Concerning ACSS2 was not a crotonyl‐transferase, several known crotonyl‐transferases were tested, which revealed that CBP and P300 exhibited the highest enzymatic activity for H4K12cr in intestinal epithelial cells (Figure S5A,B, Supporting Information). Moreover, CBP and P300 could increase H4K12cr and CLDN7 expression levels in control cells, but not in ACSS2‐knockdown cells (Figure S5C,D, Supporting Information), indicating CBP and P300 were important crotonylation “writers” for ACSS2‐mediated H4K12cr. To identify decrotonylases (including HDAC and SIRT protein families) responsible for H4K12cr “eraser”, cells were treated with TSA (HDAC inhibitor) and NAM (SIRT inhibitor), which indicated that HDAC family contributed to the removal of H4K12cr (Figure S5E–H, Supporting Information).

To further clarify the direct regulatory effect of H4K12cr on CLDN7 expression, ChIP‐seq was conducted in control and ACSS2‐knockdown NCM460 cells. A large proportion (82.8%) of peaks detected were lost in ACSS2‐knockdown cells (Figure 4F,G), mainly in the promoter regions (Figure 4H). Integration of ChIP‐seq with RNA‐seq data indicated that genes with higher expression levels possessed higher H4K12cr modification around TSS regions (Figure 4I). ACSS2‐knockdown mediated peak loss was enriched in biological functions including “positive regulation of transcription from RNA polymerase II promoter”, “focal adhesion”, and “tight junction”, implying that H4K12cr might regulate transcription of genes involved in intestinal barrier function (Figure S6, Supporting Information). Upon peaks visualization, a notable reduction of H4K12cr levels in the promoter and CDS region of *CLDN7* was observed. ATAC‐seq and RNA polymerase II ChIP‐seq also exhibited a remarkable decrease in chromatin accessibility and gene transcription of *CLDN7* (Figure 4J). Moreover, ACSS2‐mediated H4K12cr modification in *CLDN7* was validated by ChIP‐qPCR (Figure 4K,L), suggesting that CLDN7 expression is regulated by ACSS2‐mediated H4K12cr.

### 2.5 Sodium Crotonate Strengthens Intestinal Barrier Function to Alleviate Colitis

**Figure 5:**
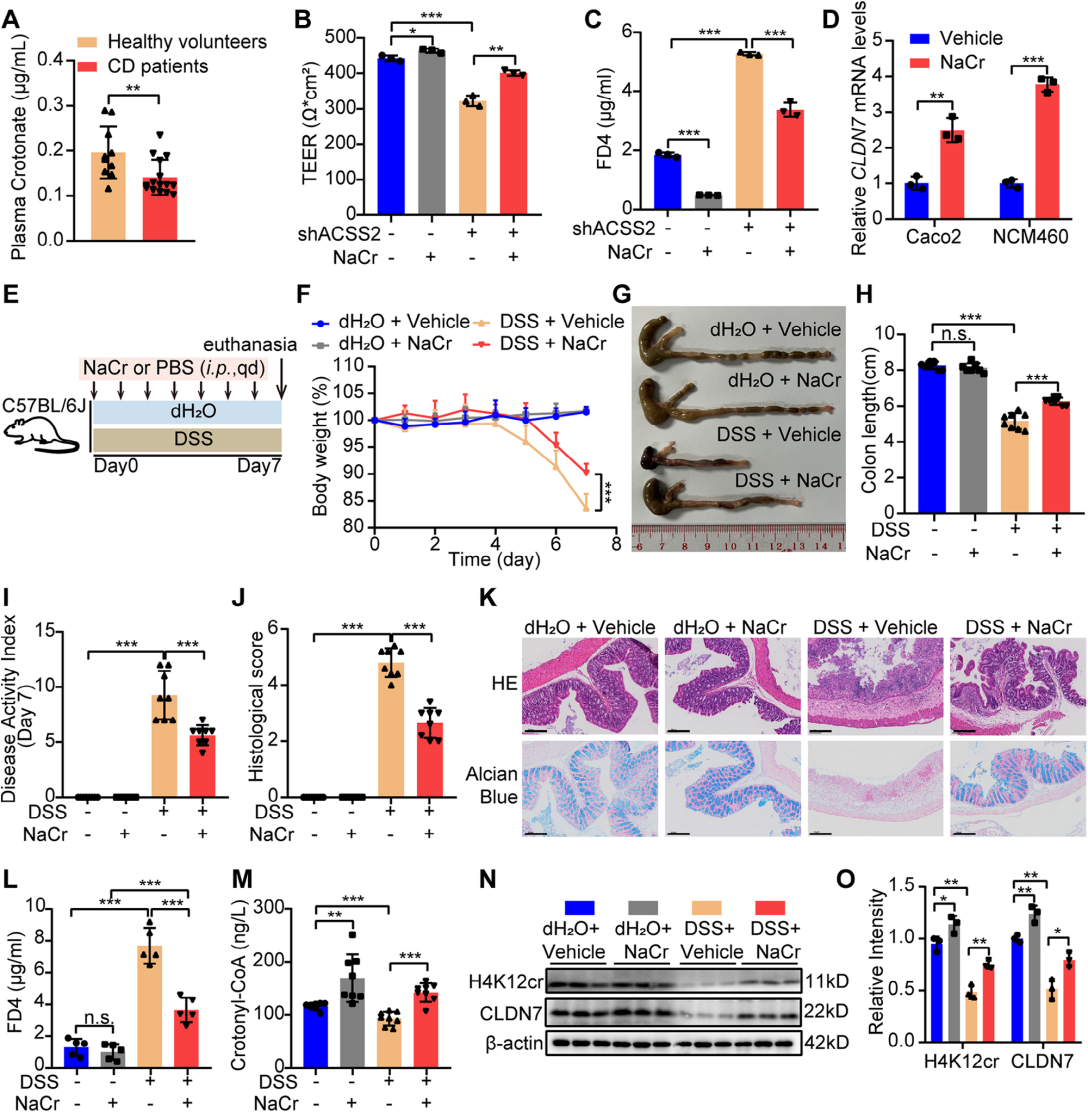
Sodium crotonate improves intestinal barrier function and ameliorates colitis. A) The plasma crotonate level in healthy volunteers (*n* = 10) and CD patients (*n* = 15), as detected by mass spectrometry. B‐C) TEER and FD4 measurement in monolayers of Caco2 cells with indicated treatment. D) Relative mRNA levels of *CLDN7* in Caco2 and NCM460 cells treated with NaCr (10 mmol L^−1^). E) The administrative strategy for mice from indicated groups. F–O) NaCr treatment alleviated colitis progression (*n* = 8 for each group). F) The mice body weight curves from indicated experimental groups. G, H) Representative colon images and colon length statistics from each group. I) The DAI statistics for mice from indicated groups. J,K) Histological score and representative HE/Alcian blue staining images of colon tissues from indicated groups. Scale bar = 100 µm. L) The plasma FD4 level in mice from indicated experimental groups. M) The plasma crotonyl‐CoA concentration in mice from indicated experimental groups. N, O) Immunoblotting and statistical analysis of H4K12cr and CLDN7 in colon tissues from indicated groups. The concentration of NaCr used in vivo was 20 mg kg^−1^ d^−1^. Values are mean ± SD. n.s. (not significant, *p* > 0.05), *p < 0.05, **p < 0.01, ***p < 0.001, determined by two‐tailed Student's *t*‐test A, D), one‐way ANOVA with Bonferroni's post hoc test B, C, H‐J, L, M, O) and repeated measures ANOVA with Bonferroni's post hoc test F).

Given crotonate is the source of crotonyl‐CoA and crotonate supplement has been validated to increase crotonyl‐CoA level and lysine crotonylation,^[^ ^22^, ^26^, ^39^ ^]^ we hypothesized that crotonate administration might enhance intestinal barrier function to alleviate colitis progression. Mass spectrometry metabolomic analysis results revealed a reduction of plasma crotonate level in CD patients (**Figure** **5**A). To delve the direct effect of crotonate, a gradient concentration of sodium crotonate (NaCr) was added into intestinal epithelial cells. NaCr could not only promote cell growth in a concentration‐dependent manner (Figure S7A,B, Supporting Information), but also rescue intestinal barrier function compromised by ACSS2 knockdown (Figure 5B,C), or inflammatory cytokines stimulation (Figure S7C–E, Supporting Information). Furthermore, NaCr treatment significantly elevated *CLDN7* expression in Caco2 and NCM460 cells (Figure 5D), suggesting that NaCr could enhance barrier function in vitro.

To evaluate the barrier‐protective effects of NaCr in vivo, a therapeutic dose previously established as safe^[^ ^39^, ^40^, ^41^ ^]^ was administered to the DSS‐induced colitis model. NaCr treatment significantly alleviated colitis symptoms and enhanced intestinal barrier function without significant adverse effects on the control mice (Figure 5E–O). Moreover, the protective effect was more profound with the increasing concentrations of NaCr administration (Figure S7F–M, Supporting Information). Additionally, plasma crotonyl‐CoA levels, as well as H4K12cr and CLDN7 protein levels in intestinal tissues were remarkably augmented following NaCr administration (Figure 5M–O). These findings collectively indicate that crotonate treatment could strengthen intestinal barrier integrity and ameliorate colitis.

### 2.6 Sodium Crotonate Alleviates Colitis Dependent on the Enzymatic Activity of ACSS2

To determine whether the therapeutic effects of NaCr in alleviating colitis are dependent on ACSS2, *Acss2* ^CKO^ mice with colitis were treated with NaCr. Interestingly, NaCr could alleviate colitis symptoms (Figure S8A–G, Supporting Information), increase plasma crotonyl‐CoA level (Figure S8H, Supporting Information) and upregulate H4K12cr and CLDN7 protein levels in *Acss2^fl/fl^* mice (Figure S8I,J, Supporting Information), but exhibited no obvious effect on *Acss2* ^CKO^ mice (Figure S8, Supporting Information). Furthermore, VY‐3‐135 treatment blocked the therapeutic effect of NaCr in vivo (Figure S9, Supporting Information). In conclusion, crotonate alleviates colitis in an ACSS2 enzymatic‐dependent manner.

### 2.7 TNF‐α Promotes the Decay of *ACSS2* mRNA by Increasing m6A Methylation

**Figure 6:**
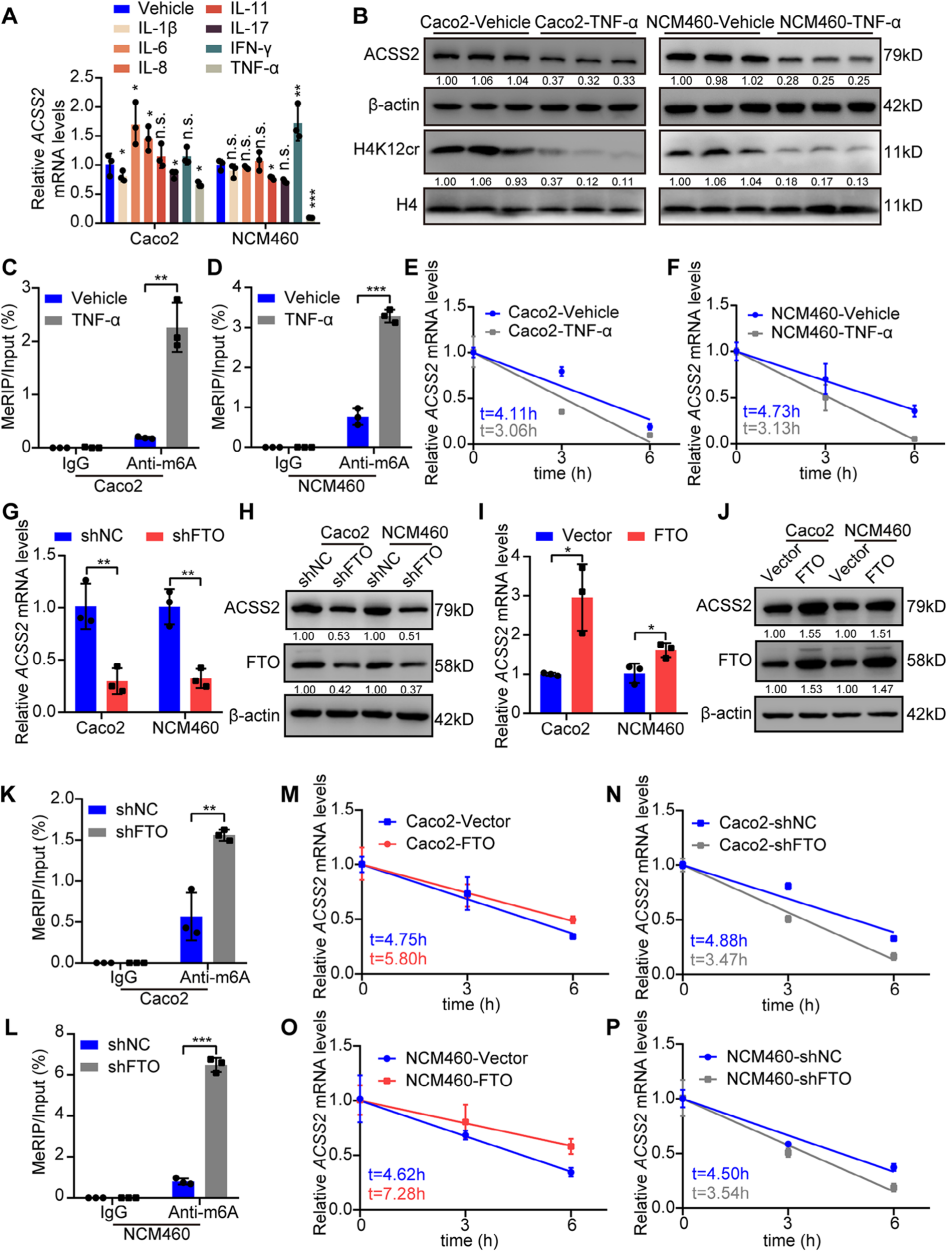
TNF‐α promotes decay of *ACSS2* mRNA by increasing its m6A methylation. A) Relative mRNA levels of *ACSS2* in Caco2 and NCM460 with indicated treatment (The concentration of all cytokines was 50 ng mL^−1^). B) Immunoblotting of H4K12cr and ACSS2 in Caco2 and NCM460 cells treated with TNF‐α. C, D) The m6A modification levels of *ACSS2* mRNA in Caco2 and NCM460 cells treated with TNF‐α. E, F) Relative mRNA levels of *ACSS2* in cells with indicated treatment, collected in indicated timepoint. The half‐life was calculated by the fitted line. G, H) FTO knockdown decreased ACSS2 expression in Caco2 and NCM460 cells, as detected by G) qRT‐PCR and H) immunoblotting. I, J) FTO overexpression enhanced ACSS2 expression in Caco2 and NCM460, as detected by I) qRT‐PCR and J) immunoblotting. K,L) The m6A modification level of *ACSS2* mRNA in control and FTO‐knockdown Caco2 and NCM460 cells. M‐P) Relative mRNA levels of *ACSS2* in control, FTO‐knockdown, and FTO‐overexpression Caco2 and NCM460 cells at indicated timepoint. The half‐life was calculated by the fitted line. Relative band intensities are annotated below respective lanes (B, H, J). Values are mean ± SD. n.s. (not significant, *p* > 0.05), *p < 0.05, **p < 0.01, ***p < 0.001, determined by one‐way ANOVA with Bonferroni's post hoc test A), two‐tailed Student's *t*‐test C, D, G, I, K, L).

Inflammatory cytokines are known to contribute to IBD progression by reprogramming gene expression in intestinal epithelium.^[^ ^42^, ^43^, ^44^ ^]^ To reveal the mechanism behind *ACSS2* downregulation, we treated intestinal epithelial cells with a panel of inflammatory cytokines. Tumor necrosis factor‐alpha (TNF‐α) remarkably decreased *ACSS2* mRNA levels compared to other cytokines (**Figure** **6**A). Moreover, the protein levels of ACSS2 and H4K12cr were also reduced upon TNF‐α treatment (Figure 6B). Previous studies underscored a crucial post‐transcriptional regulatory manner, N6‐methyladenosine (m6A) modification could be controlled by TNF‐α.^[^ ^45^, ^46^ ^]^ Therefore, MeRIP assays were conducted and revealed an increase of m6A levels in *ACSS2* mRNA (Figure 6C,D) in response to TNF‐α treatment. Moreover, *ACSS2* mRNA stability was declined following TNF‐α treatment (Figure 6E,F). The m6A modification level is precisely regulated by m6A methylases or demethylases. To identify the key enzyme responsible for m6A modification in *ACSS2* mRNA upon TNF‐α treatment, the involved enzymes were evaluated in TNF‐α‐treated cells. Among all m6A‐associated enzymes, FTO was downregulated most significantly in TNF‐α‐treated cells (Figure S10, Supporting Information). Furthermore, FTO knockdown obviously decreased ACSS2 expression (Figure 6G,H), while FTO overexpression increased ACSS2 expression (Figure 6I,J). Besides, the m6A level of *ACSS2* mRNA was significantly elevated in cells with FTO knockdown (Figure 6K,L), while the stability of *ACSS2* mRNA was increased in FTO‐overexpressed cells or decreased in FTO‐knockdown cells (Figure 6M–P). These results suggest that TNF‐α downregulates FTO expression to enhance m6A modification of *ACSS2* mRNA, thereby inhibiting its mRNA stability and expression.

### 2.8 Anti‐TNF‐α Therapy Enhances the Therapeutic Effect of Sodium Crotonate on Colitis

**Figure 7:**
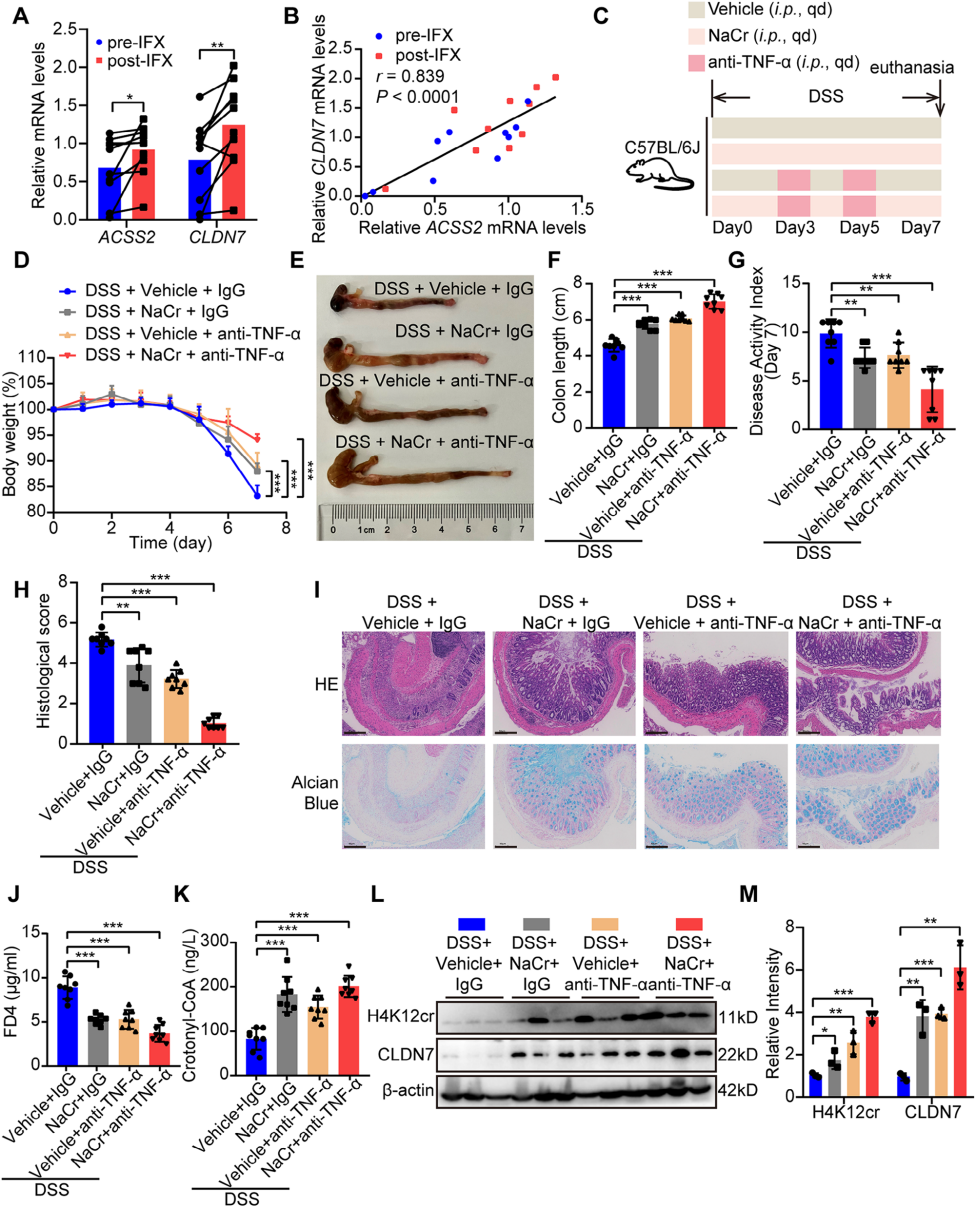
Anti‐TNF‐α therapy enhances the therapeutic effect of NaCr on colitis. A) Relative mRNA levels of *ACSS2* and *CLDN7* in ten pairs of intestinal biopsies from CD patients before and after IFX treatment. B) Correlation analysis results of relative mRNA levels of *ACSS2* and *CLDN7*. C) The administrative strategy of anti‐TNF‐α therapy and NaCr in DSS‐induced colitis models. D‐L) Combining anti‐TNF‐α therapy and NaCr significantly alleviated colitis progression (*n* = 8 for each group). D) The mice body weight curves from indicated experimental groups. E, F) Representative colon images and colon length statistics from each group. G) The DAI statistics for mice from the indicated groups. H, I) Histological score and representative HE/Alcian blue staining images of colon tissues from indicated groups. Scale bar = 100 µm. J) The plasma FD4 level in mice from the indicated groups. K) The plasma crotonyl‐CoA level in mice from the indicated groups. L‐M) Immunoblotting and statistical analysis of H4K12cr and CLDN7 in colon tissues from the indicated groups. Values are mean ± SD. *p < 0.05, **p < 0.01, ***p < 0.001, determined by two‐tailed Student's *t*‐test A), Pearson's correlation with two‐tailed test B), repeated measures ANOVA with Bonferroni's post hoc test D), and one‐way ANOVA with Bonferroni's post hoc test F‐H, J, K, M).

As ACSS2 was downregulated by TNF‐α, we hypothesized that anti‐TNF‐α therapy might polish up the barrier‐protective effect of NaCr. We primarily collected ten pairs of intestinal mucosal biopsies from CD patients before and after infliximab (IFX) administration to delve the impact of IFX treatment on ACSS2 and CLDN7 expression. These results indicated that *ACSS2* and *CLDN7* mRNA expression were significantly increased after IFX treatment (**Figure** **7**A), along with a positive correlation between *ACSS2* and *CLDN7* mRNA levels (Figure 7B). In murine colitis models, the combination of anti‐TNF‐α therapy with NaCr significantly alleviated colitis progression (Figure 7C–I), improved intestinal barrier function (Figure 7J) and enhanced plasma crotonyl‐CoA production compared to NaCr treatment alone (Figure 7K–M). These findings collectively suggest that anti‐TNF‐α therapy enhances the therapeutic effect of NaCr on colitis by upregulating ACSS2 and CLDN7 expression.

## 3 Discussion

**Figure 8:**
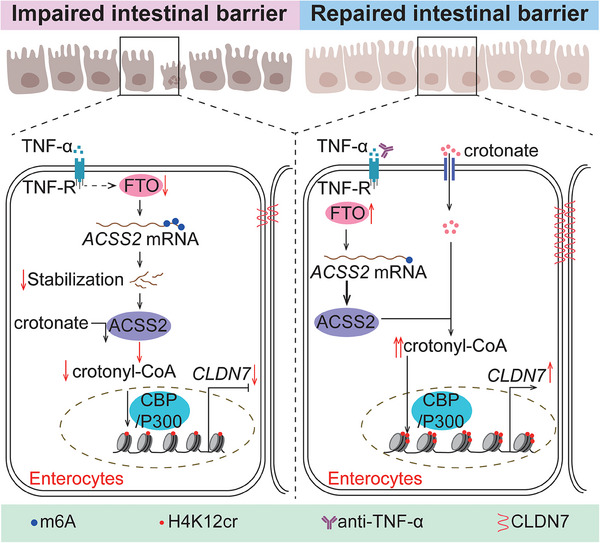
Schematic diagram revealing the critical role of ACSS2‐H4K12cr‐CLDN7 axis in maintaining intestinal barrier function. In inflamed intestinal epithelium, TNF‐α reduces FTO expression, thereby destabilizing and decreasing *ACSS2* mRNA by increasing its m6A modification. Reduced ACSS2 diminishes H4K12cr, leading to *CLDN7* downregulation and intestinal barrier destruction. Combining anti‐TNF‐α therapy with crotonate supplementation increases ACSS2 and CLDN7 expression and repair intestinal barrier function.

PTMs, particularly histone modifications play crucial roles in IBD pathogenesis by maintaining the intestinal epithelial barrier.^[^ ^12^, ^13^, ^14^ ^]^ As a novel PTM type, protein crotonylation's functions in IBD progression remain largely unexplored. Our study identifies a key regulator for protein crotonylation, ACSS2, which was downregulated in the inflamed intestinal epithelium and demonstrated a negative correlation with colitis progression. Genetic or pharmacologic inhibition of ACSS2 impaired intestinal barrier function by decreasing CLDN7 expression. Mechanistically, ACSS2‐mediated H4K12cr upregulated the transcription of *CLDN7*, which could be strengthened by crotonate supplement. Additionally, TNF‐α was found to reduce ACSS2 expression via enhancing the m6A modification of *ACSS2* mRNA. Overall, our findings demonstrate ACSS2‐CLDN7 axis as an attractive therapeutic target for intestinal epithelial barrier repair and colitis alleviation (Figure 8).

As a key enzyme responsible for crotonyl‐CoA production, ACSS2 has been implicated in kidney fibrosis^[^ ^27^ ^]^ and stem cell osteogenesis^[^ ^47^ ^]^ via regulating protein crotonylation. Previous research has indicated that ACSS2 is required for butyrate‐dependent Treg differentiation to mitigate colitis.^[^ ^30^ ^]^ Herein, we revealed that ACSS2‐mediated histone Kcr was indispensable for intestinal epithelial barrier function, for the first time. It's noteworthy that ACSS2 could also modulate histone lysine acetylation^[^ ^37^, ^38^ ^]^ or butyrylation.^[^ ^30^ ^]^ Compared with Pan‐Kac and Pan‐Kbu, the Pan‐Kcr level decreased most profoundly following ACSS2 knockdown in intestinal epithelial cells. Moreover, epithelial barrier function was particularly responsive to crotonyl‐CoA supplementation compared to acetyl‐CoA and butyryl‐CoA, indicating the involvement of crotonylation in ACSS2‐mediated barrier protection. However, these PTMs share similar enzyme systems,^[^ ^18^, ^48^ ^]^ making it challengeable to delineate the specific contribution of ACSS2‐mediated crotonylation to barrier protection. Further investigations are needed to identify the separate effect of each PTM utilizing advanced methodologies.

Previous studies have shown that Pan‐Kcr modifications are significantly stronger in brain and colon tissues compared to other organs such as liver, spleen, and kidneys.^[^ ^49^ ^]^ Based on these tissue‐specific observations, our study further revealed that the intestinal epithelium may serve as a major source of crotonyl‐CoA in the intestine. Notably, we extended these findings to systemic circulation by detecting plasma crotonyl‐CoA levels. Intriguingly, plasma crotonyl‐CoA levels moderately decreased during intestinal inflammation, and could be restored by either NaCr supplementation or anti‐TNF‐α treatment. These results collectively suggest that circulating crotonyl‐CoA levels may serve as a potential biomarker for predicting both the onset and therapeutic response of intestinal inflammation.

CLDN7, essential for intestinal epithelial homeostasis,^[^ ^34^, ^35^ ^]^ tight junction assembly, and intestinal barrier destruction,^[^ ^7^, ^36^ ^]^ was frequently decreased in the inflamed intestine. However, how CLDN7 was downregulated was poorly understood. Histone crotonylation predominantly enriches in promoter and CDS regions, activating gene transcription.^[^ ^21^, ^22^, ^23^ ^]^ For example, Histone H4 lysine crotonylation could enhance the expression of immunogenic retroelements to elicit anti‐tumor immune response.^[^ ^22^ ^]^ Similarly, both H3K9cr and H3K27cr have been shown to promote gene expression.^[^ ^26^, ^27^ ^]^ Our ChIP‐seq and ATAC‐seq data suggest that ACSS2‐mediated H4K12cr enhances CLDN7 expression by increasing chromatin accessibility and transcriptional activity in intestinal epithelium. These results pinpointed the pivotal correlation between histone Kcr and CLDN7 expression. Furthermore, we also identified CBP and P300 as key regulators of H4K12cr modification and CLDN7 expression, emphasizing the indispensable function of histone Kcr in regulating intestinal epithelial barrier integrity. Given the intricate regulatory network and the diversity of modified residues in histones, more investigations are required to reveal the involvement of other histone Kcr modifications in IBD progression.

During the pathogenesis and progression of IBD, significant alterations occur in the composition of gut microbiota, which exert crucial impacts on cellular functions within the intestinal microenvironment and even systemic metabolism.^[^ ^50^, ^51^, ^52^, ^53^ ^]^ Our findings demonstrated a substantial reduction in the diversity of gut microbiota under colitis conditions. Specifically, *Bacillota* and *Bacteroidota* were the two types of bacteria with the highest abundance, and both underwent significant abundance changes during colitis in *ACSS2* ^CKO^ mice. This implied that the observed changes in these bacteria might lead to decreased production of crotonate in vivo, thereby partially aggravating colitis progression. However, our study had not revealed specific bacterial species that directly regulate crotonate production and colitis progression. Therefore, future research should focus on elucidating the precise roles of gut microbiota.

As the substrate for crotonylation, crotonate has been previously shown to strengthen protein crotonylation.^[^ ^22^, ^39^ ^]^ Due to the relatively low concentration in plasma, the functional role of crotonate is largely underestimated in IBD. Our results indicated a decrease of crotonate in plasma from IBD patients, and crotonate supplementation effectively alleviated experimental colitis via the ACSS2‐H4K12cr‐CLDN7 axis. These results highlight that crotonate administration could be a convenient and effective strategy for colitis treatment, but this approach needs further cohort studies to validate its safety and efficacy for clinical practice. Interestingly, crotonate level was also decreased in serum from psoriasis patients, which emerged as an essential biomarker associated with psoriasis pathogenesis.^[^ ^54^ ^]^ Therefore, crotonate may function as a biomarker for disease prediction or prognosis estimation in IBD, and even other auto‐immune diseases.

TNF‐α, a well‐known pro‐inflammatory cytokine, was implicated in IBD progression through various mechanisms.^[^ ^55^, ^56^, ^57^ ^]^ Herein, our study demonstrated that TNF‐α inhibits FTO to destabilize *ACSS2* mRNA by increasing its m6A modification, leading to a decline of histone Kcr levels. Similarly, TNF‐α has been found to reduce gene expression in other contexts via promoting m6A modification.^[^ ^45^, ^46^ ^]^ In our CD cohort, IFX was uncovered to relieve ACSS2 and CLDN7 downregulation. Moreover, combining anti‐TNF‐α therapy and therapeutic manipulation of crotonylation significantly alleviated colitis and intestinal barrier function, suggesting this might serve as a promising treatment approach to IBD.

However, there are some limitations to our study. The extent to which the barrier‐protection effect of ACSS2 depends on crotonylation remains challenging to characterize, even though we observed that barrier function was more sensitive to crotonylation. Additionally, the presence of concurrent crotonylated residues in histones raises questions about the potential importance of other forms of histone Kcr in colitis progression. Finally, further real‐world studies are required to assess the safety as well as therapeutic effects of crotonate in IBD.

In conclusion, our study highlights the critical role of histone crotonylation patterns in intestinal epithelium from IBD, underscoring the essential role of ACSS2‐mediated H4K12cr in CLDN7 transcription and intestinal barrier function. These findings collectively imply the potential therapeutic effects of crotonate in IBD patients.

## 4 Experimental Section

### Patients and Clinical Specimens

Paraffin‐embedded colon tissues from CD patients (*n* = 24) and UC patients (*n* = 9) were collected for immunohistochemistry (IHC) staining. The clinicopathologic information of IHC cohort is provided in Table S1 (Supporting Information). For *ACSS2* and *CLDN7* mRNA expression detection, intestinal biopsies from CD patients (*n* = 26) were processed for quantitative real time‐qPCR, of which ten CD patients with paired biopsies before and after IFX treatment were used for mRNA expression changes upon IFX treatment. The clinicopathological characteristics are listed in Table S2 (Supporting Information). Adjacent normal colon tissues (*n* = 25 in IHC, *n* = 26 in qRT‐PCR) resected during colectomy served as healthy controls. Human plasma samples were obtained from ten healthy volunteers and CD patients for crotonate (*n* = 15) and crotonyl‐CoA detection (*n* = 28). Detailed clinicopathologic information of IBD patients is given in Table S3 (Supporting Information). Patients or the public were not involved in the design, conduct, reporting, or dissemination plans of our research. Specimens’ collection with informed consent was carried out in the Sixth Affiliated Hospital of Sun Yat‐sen University, approved by the Institutional Review Board of the Sixth Affiliated Hospital of Sun Yat‐sen University (2024ZSLYEC‐261).

### Bioinformatic Analysis

The GSE59071 dataset was acquired from gene expression omnibus. Single‐cell transcriptional sequence data from the study conducted by Smillie et al. was re‐analyzed and visualized in the scIBD platform (http://scibd.cn/#).

### Acyl‐Co‐Enzyme A Concentration Measurement

The intracellular concentration of acyl‐CoA was quantitatively determined using ultra‐performance liquid chromatography‐tandem mass spectrometry (UPLC‐MS/MS). Briefly, cell samples were rapidly quenched in liquid nitrogen for 10 min to arrest metabolic activity, followed by the addition of 600 µL of pre‐chilled methanol (−20 °C). The extraction process was enhanced by the addition of chloroform, with subsequent vortexing at 1400 rpm for 1 min. To facilitate phase separation, ultra‐pure water was added, and the mixture was maintained on ice for 10 min. Then the samples were centrifuged at 4 °C and 12 000 rpm for 2 min. The supernatant was collected and dried using a vacuum concentrator. Both the processed samples and acyl‐CoA standard solutions were reconstituted in methanol for UPLC‐MS/MS. Quantification of intracellular acyl‐CoA was performed by comparing the sample signals with a standard calibration curve established by acyl‐CoA standards. The extracellular crotonyl‐CoA in human and mouse plasma was assessed by the enzyme‐linked immunosorbent assay (ELISA) Kit (#MM‐51450H2 or #MM‐44933M2) following the manufacturer's instructions. After antigen crosslinking and chromogenic reaction, the OD values of plates were measured at a wavelength of 450 nm.

### RNA Isolation and qRT‐PCR

Total RNA was isolated using TRIzol Reagent (Invitrogen, CA, USA) and reverse transcribed into cDNA utilizing the ReverTra Ace qPCR RT Kit (Toyobo, Japan). Quantitative real‐time PCR (qRT‐PCR) was conducted using ABI QuantStudio™ 7 RealTime PCR Systems. The primers used are provided in Table S4 (Supporting Information). The relative expression levels of target genes were normalized by β‐actin utilizing the 2^‐ΔΔCt^ method.

### Immunoblotting

RIPA lysis buffer (Beyotime, Shanghai, China) containing protease and phosphatase inhibitors (Thermo Fisher Scientific, USA) was utilized for protein extraction. Protein samples were processed for SDS‐PAGE and transferred to PVDF membranes (Millipore, Massachusetts, USA). Blocked by 5% skim milk, the PVDF membranes were incubated with indicated antibodies overnight (listed in Table S5, Supporting Information). The next day, membranes were incubated with secondary antibody at room temperature for 1 h after TBST washing. Finally, the membranes were exposed by ECL reagents.

### Immunohistochemistry

Paraffin‐embedded slides were primarily placed at 65 °C for 4h. Following de‐paraffinizing, slides were rehydrated and immersed in EDTA buffer for antigen retrieval. Later, slides were blocked with 2.5% goat serum and incubated with specific primary antibodies (listed in Table S5, Supporting Information) overnight. Subsequently, slides were washed with PBS and the corresponding secondary antibody was added onto the slides. The proteins were stained by diaminobenzidine. The protein levels were determined by two independent pathologists following the score system previously described.^[^ ^58^ ^]^

### Mice

All mice were raised in the Sixth Affiliated Hospital of Sun Yat‐Sen University and Guangzhou Ruige Biological Technology Co., Ltd., approved by the Institutional Animal Care and Use Committee (IACUC) at the Sun Yat‐Sen University (IACUC‐2023082501 and IACUC‐2023102602). C57BL/6J wild‐type mice, mice on a C57BL/6JGpt background carrying *Flox*‐flanked alleles of *Acss2* (*Acss2^fl/fl^*) and *Vil1*‐*cre* mice were obtained from Gempharmatech Co. Ltd. (Jiangsu and Guangdong, China). *Acss2^fl/fl^* mice crossed with *Vil1*‐*cre* mice to generate mice with intestinal epithelial *Acss2* conditional knockout (*Vil1‐cre*; *Acss2^fl/fl^*; termed as *Acss2* ^CKO^). Mice lived in specific pathogen‐free facilities with a 12‐h light‐dark cycle. Primers used for mice genotyping are provided in Table S6 (Supporting Information).

### Dextran Sulfate Sodium Salt (DSS)‐Induced Colitis

To establish experimental colitis, mice (6 to 8‐week‐old, male, weighing 20‐28g) were included and randomly assigned to colitis groups or control groups, using a computer‐based random order generator. Mice were fed with DSS solution (2.5%, MP Biomedicals, USA) in water for continuous 7 days. The control groups received normal drinking water. Experimental methods and results were shown following the ARRIVE reporting guidelines.^[^ ^59^ ^]^ Mice were daily monitored with body weight, stool consistency, and rectal bleeding to calculate disease activity index (DAI) score, the monitor time point was constant to exclude the effect of biological rhythm on measurement. During colitis induction, NaCr solution (5, 10, 20 mg kg^−1^, intraperitoneal injection), VY‐3‐135 (50 mg kg^−1^, intraperitoneal injection), anti‐TNF‐α antibody (10 mg kg^−1^, intraperitoneal injection, BioXcell, USA, #BE0244) or IgG isotype control (10 mg kg^−1^, intraperitoneal injection, BioXcell, USA, #BE0091) were administrated to mice in the respective experiment. On the 8th day, indicated mice were practiced euthanasia to obtain intestine and plasma for histopathological analysis, intestinal permeability detection, and crotonyl‐CoA measurement. Mice with failed colitis induction were excluded from the analysis. Detailed guidelines for DAI score and histopathological analysis methods have been described previously.^[^ ^60^ ^]^

### Intestinal Permeability Detection

For intestinal permeability assessment, mice were fasted for 8 h, followed by oral administration of fluorescein isothiocyanate (FITC)‐labeled dextran (0.6 mg g^−1^ body weight, FD40000, MedChemExpress, Shanghai, China, #HY‐128868D). Four hours after gavage, mice were sacrificed, and plasma samples were collected for fluorescence intensity measurement at a wavelength of 490 nm. The standard curve of FD40000 was utilized for concentration calculation.

### High Throughput 16S rRNA Amplicon Sequencing and Analysis

Fecal DNA was extracted by FastDNA Spin Kit for Soil (MP Biomedicals). Barcoded amplicons targeting the V3‐V4 region were produced via PCR. Firstly, 10 ng of genomic DNA was processed for the PCR using the 338F (5'‐ACTCCTACGGGAGGCAGCAG‐3') and 806R (5'‐GGACTACHVGGGTWTCTAAT‐3') primers. Following amplification, PCR products were purified and quantified. These products were then multiplexed in equimolar amounts and sequenced on an Illumina MiSeq PE300 platform (2 × 300 bp). Microbial analysis was conducted by Quantitative Insights into Microbial Ecology 2 (QIIME2) software. Reads were imported, quality‐filtered, demultiplexed, and classified against the Greengenes 16S rRNA gene database. Shannon index analysis and principal coordinate analysis (PCoA) were carried out using R software (version 4.1.2). 16S rRNA data are deposited in NCBI with accession codes PRJNA1248129.

### Cell Culture and Treatment Reagents

The NCM460, Caco2 and HEK293T cells were acquired from American Type Culture Collection, cultured in DMEM (Gibco, USA) containing 1% streptomycin–penicillin (Gibco, USA) and 10% fetal bovine serum (FBS; Gibco, USA), and incubated at 37 °C in an atmosphere of 5% CO_2_. Mycoplasma contamination was examined monthly to qualify cells. All reagents used are listed in Table S7 (Supporting Information). To evaluate the barrier‐protective effects of acyl‐CoA, Caco2 cells were treated with the indicated acyl‐CoA every other day until the successful establishment of the cell barrier model in the TEER and FD4 assay. Crotonate, premixed with water, was adjusted the pH value to 7.0 by sodium hydroxide solution to generate sodium crotonate (NaCr) solution for in vitro and in vivo usage.

### Plasmids, Transient Infection, and Lentiviral Transduction

Primers used for plasmid construction are listed in Table S8 (Supporting Information). To construct plasmids for ACSS2 and FTO knockdown, short hairpin RNA sequences were designed and inserted into pLKO.1 lentiviral vector (Addgene). The source of plasmids for gene overexpression are provided in Table S9 (Supporting Information). Lipofectamine® 3000 was employed for transient infection according to the instructions. Packaging plasmids (psPAX2 and pMD2.G; Addgene) for stable infection, were premixed with target plasmids and polyethylenimine (Polysciences, #24765‐100), introducing into HEK293T cells. Cells were incubated in supernatant of HEK293T containing lentivirus for 2 days and selected with indicated antibiotics to ensure stable transfection.

### RNA Sequencing

NCM460 with control or ACSS2 knockdown were processed for total RNA extraction. RNA samples were purified and fragmented before constructing cDNA library. Then the libraries were sequenced and aligned to reference genome by Bowtie2 software. Differentially expressed genes were analyzed by DESeq2 packages.

### Transepithelial Electrical Resistance (TEER) Measurement and FD4000 Paracellular Permeability Detection

Caco2 cells (2 × 10^5^) with different transfection were cultured in the upper chamber of transwell (Corning, New York, USA, #3401) for 20 days, with medium refreshed every 2 days, until forming a monolayer barrier. The successful construction of the cell barrier model was confirmed by a TEER measurement of over 300 Ω×cm^2^. Following the indicated treatment, TEER was measured using a Millicell ERS‐2 Voltohmmeter (Millipore, Darmstadt, Germany).

For paracellular permeability measurement, fluorescein isothiocyanate (FITC)‐labeled dextran (MW 4000, FD4000, MedChemExpress, Shanghai, China, #HY‐128868A) was diluted in Opti‐MEM medium to a concentration of 1 mg mL^−1^. The medium was replaced with 300 µL of diluted FITC‐dextran in the upper chamber and 800 µL of pure Opti‐MEM medium in the lower compartment. After incubating at 37 °C for 4 h, 200 µL medium in the lower compartment was extracted for fluorescence intensity detection at a wavelength of 490 nm. The standard curve of FD4000 was utilized for dextran content calculation.

### Adeno‐Associated Virus (AAV) Production and Infection

AAV9 vectors for overexpression of mouse *Cldn7* overexpression specifically in intestinal epithelium were constructed using the vector (pAAV9‐vil promoter‐MCS‐EGFP‐SV40‐PolyA), produced by Genechem Co., Ltd. (Shanghai, China). For in vivo infection, AAV9 or control virus particles (1 × 10^11^ physical particles per mouse) were administered to indicated mice (4 weeks of age) by intraperitoneal injection. Four weeks after injection, mice were prepared for colitis induction.

### Immunoprecipitation and Mass Spectrometry

To detect endogenous protein modified by lysine crotonylation, cells were lysed in immunoprecipitation lysis buffer at 4 °C for 30 min, and then subjected to centrifugation for protein supernatant. Prewashed protein A/G magnetic beads (Thermo Fisher Scientific, St Peters, MO, USA) were mixed with anti‐Pan‐Kcr (PTMBIO, #PTM‐501) to crosslink for 4 h at 4 °C and then added into the protein lysate for enrichment at 4 °C. The next day, the proteins absorbed in magnetic beads were eluted by 2× SDS loading buffer at 100 °C and processed for SDS‐PAGE. The gel lane was cut for mass spectrometry to identify proteins. The endogenous proteins modified by lysine crotonylation are listed in Table S10 (Supporting Information).

### Chromatin Immunoprecipitation (ChIP) ‐seq and ChIP‐qPCR

ChIP was performed following the instructions of the ChIP assay kit (Sigma‐Aldrich, USA, #17–10085). Indicated cells were crosslinked by formaldehyde and quenched with glycine solution. After fragmented, cell lysates were incubated with antibodies against H4K12cr (PTMBIO, #PTM‐530), RNA pol II (Abcam, #ab5408) or negative control mouse IgG (Proteintech, #B900620) and protein A/G magnetic beads at 4 °C for 12 h. Subsequently, chromatin fragments modified with H4K12cr were eluted and purified for ChIP‐seq or ChIP‐qPCR.

For ChIP‐seq analysis, raw reads in fastq format were cleaned by removing adapters and low‐quality reads using fastp software. Then clean reads were mapped to human reference genome by Bowtie2 software, followed by PCR duplication removal and peak calling. Peaks detection and differentially accessible peaks were obtained using MACS2 software. Deeptools software was used to visualize the signal strength of H4K12cr signal (±5 kb around Transcriptional Start Site). Peak strength around the *CLDN7* gene was visualized by IGV software. The ChIPseeker package was used to annotate peaks and genome region distribution. GO and KEGG analysis were conducted by ClusterProfiler. The H4K12cr ChIP‐qPCR data were presented as fold changes compared to the IgG group, and the primers for the *CLDN7* promoter region were as follows: Forward: 5'‐GGGGCGCACCTGAGTATATG‐3' and Reverse: 5'‐ GTGAGTGTCCCTTCGGTGAC‐3'.

### Assay for Transposase Accessible Chromatin (ATAC)‐seq

The indicated cells were processed using the Hyperactive ATAC‐Seq Library Prep Kit for Illumina (Nanjing Vazyme Biotech, China, #TD711) following the instructions. Briefly, cells were lysed and fragmented with corresponding buffer. DNA fragments were then separated by magnetic beads for preliminary DNA library construction. After sorting and purification, DNA library was prepared for high‐throughput sequencing. Fastq data were cleaned by removing adapter and low‐quality reads, followed by mapping to reference genome. After PCR duplication removal, reads were processed to peak calling and visualized by IGV.

### Gas Chromatograph and Mass Spectrometry for Crotonate Detection

The concentration of plasma crotonate was analyzed by PANOMIX Biomedical Tech Co., Ltd. (Suzhou, China). Briefly, standard stock solutions of crotonate (100 mg mL^−1^) were prepared, along with a working solution series by appropriate dilutions of standard stock solution with water. An internal standard (IS) solution containing 4‐methylvaleric acid was mixed with crotonate working solution series and 15% phosphoric acid to generate a calibration curve. Human plasma was mixed in 15% phosphoric acid with the IS solution. The samples were vortexed, centrifuged, and the supernatant was transferred for GC‐MS analysis. Mass spectrometric detection of metabolites was carried out using the ISQ 7000 mass spectrometer (Thermo Fisher Scientific, USA).

### Cell Viability Assay

Briefly, 1000 cells were cultured in 96‐well plates with indicated treatment. After the specified incubation times, the original medium was replaced with culture medium containing 10% CCK‐8 substrate (Solarbio, Beijing, China, #CA1210). Cells were incubated at 37 °C for 2h and measured OD values at a wavelength of 450 nm.

### Methylated RNA Immunoprecipitation (MeRIP)‐qPCR

MeRIP was conducted by the EpiQuik CUT&RUN m6A RNA Enrichment Kit (EpigenTek, New York, USA, #P9018). Briefly, 10 µg RNA from indicated cells were immunocaptured by m6A antibody and cleaved by an enzyme mix following the manufacture's instruction. The m6A modified RNA was then eluted for MeRIP‐qPCR detection. The m6A modification site of *ACSS2* was predicted by SRAMP, and the primers for detection of *ACSS2* m6A modification level were as follows: Forward: 5'‐CTCCCTTGACCAGCTGTCTG‐3' and Reverse: 5'‐GCCCTCACATCCCCTTTGAA‐3'.

### RNA Stability Assay

Cells with indicated treatment were incubated with Actinomycin D (10 µg mL^−1^, MedChemExpress, Shanghai, China, #HY‐17559) for 0, 3, and 6 h. Following incubation, mRNA was extracted in each group and reverse transcribed into cDNA for ACSS2 expression detection. The mRNA expression level of *ACSS2* at different time points was linearly fitted, and the half‐life of *ACSS2* under different treatment conditions was calculated.

### Statistical Analysis

Statistical analysis was conducted by GraphPad Prism software (La Jolla, CA, USA). The data were derived from at least three independent experiments and shown as mean ± standard deviation (SD). The two‐tailed Student's *t*‐test was used for comparison of two independent groups and one‐way analysis of variance (ANOVA) with Bonferroni's post hoc test was performed for comparison of multiple groups. Variables from repeated measurements were analyzed by repeated measures ANOVA with Bonferroni's post hoc test. The correlation between variables was calculated by Pearson's correlation analysis. Detailed statistical information was provided in corresponding figure legends.

## Conflict of Interest

The authors declare no conflict of interest.

## Author Contributions

T.H., X.G., P.L., and X.R.W. contributed equally to this work as co‐corresponding authors. M.Y., S.P.C., Z.S.L., and R.F.Y. were co‐first authors and performed most of the experiments. K.C., S.B.Y., Q.L., H.X.K., C.Z., J.F.H., and G.Z.L. collected the clinical samples and provided technical assistance. M.Y., C.S.P., Z.S.L., and R.F.Y. conceived the project, analyzed exprerimental results, and wrote the original manuscript. T.H., X.G., P.L., and X.R.W. reviewed and edited the manuscript. All authors approved the submitted manuscript.

## Supporting information



Supporting Information

## Data Availability

The data that support the findings of this study are available from the corresponding author upon reasonable request.
