# Supplementary material for: ACSS2‐Mediated Histone H4 Lysine 12 Crotonylation (H4K12cr) Alleviates Colitis via Enhancing Transcription of CLDN7
Source: Adv Sci (Weinh). 2025 Jul 12;12(30):e00461. doi: 10.1002/advs.202500461 (PMC12376547; doi:10.1002/advs.202500461)
Supplement: Supplementary file 1 — Supporting Information [file ADVS-12-e00461-s001.pdf]

ADVANCED SCIENCE
Open Access

Supporting Information

for *Adv. Sci.*, DOI 10.1002/advs.202500461

ACSS2-Mediated Histone H4 Lysine 12 Crotonylation (H4K12cr) Alleviates Colitis via Enhancing Transcription of CLDN7

Ming Yuan, Shaopeng Chen, Zhensen Lin, Runfeng Yu, Kang Chao, Shubiao Ye, Qing Li, Haoxian Ke, Chi Zhang, Junfeng Huang, Guanzhan Liang, Tuo Hu, Xiang Gao*, Ping Lan* and Xianrui Wu**

Supporting Information

Title: ACSS2-Mediated Histone H4 Lysine 12 Crotonylation (H4K12cr) Alleviates Colitis via Enhancing Transcription of CLDN7

Ming Yuan, *Shaopeng Chen*, *Zhensen Lin*, *Runfeng Yu*, *Kang Chao*, *Shubiao Ye*, *Qing Li*, *Haoxian Ke*, *Chi Zhang*, *Junfeng Huang*, *Guanzhan Liang*, *Tuo Hu**, *Xiang Gao**, *Ping Lan**, *Xianrui Wu**

This file includes:

Figure S1 to S10

Table S1 to S10

Supplementary Figures

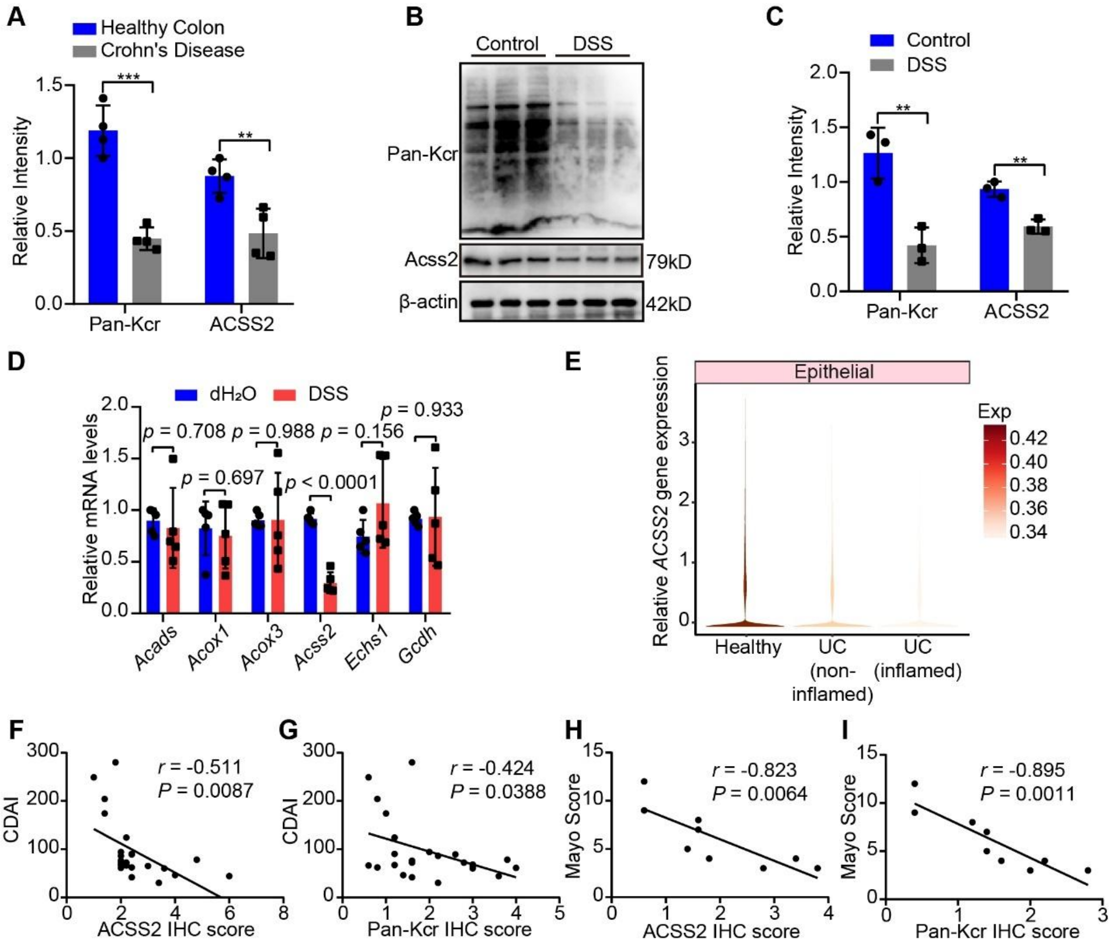

Figure S1. Decreased Pan-Kcr and ACSS2 expression in inflamed intestinal epithelium. A) Statistical analysis of relative immunoblotting intensity in colon tissues from healthy controls and CD patients. B) Immunoblotting of Pan-Kcr and ACSS2 in colon tissues from normal control and mice colitis models. C) Statistical analysis of relative immunoblotting intensity in colon tissues from normal control and murine colitis models. D) Relative mRNA levels of enzymes for crotonyl-CoA production in colons from normal control and mice colitis models (n = 5 per group). E) Relative mRNA levels of *ACSS2* in intestinal epithelial cells from single cell transcriptomics

data, analyzed by scIBD platform. F-I) Correlation analysis of indicated IHC score and disease activity index (CDAI and Mayo score). Values are mean ± SD, determined by two-tailed Student's *t*-test (A, C, D), and Pearson's correlation with two-tailed test (F-I).

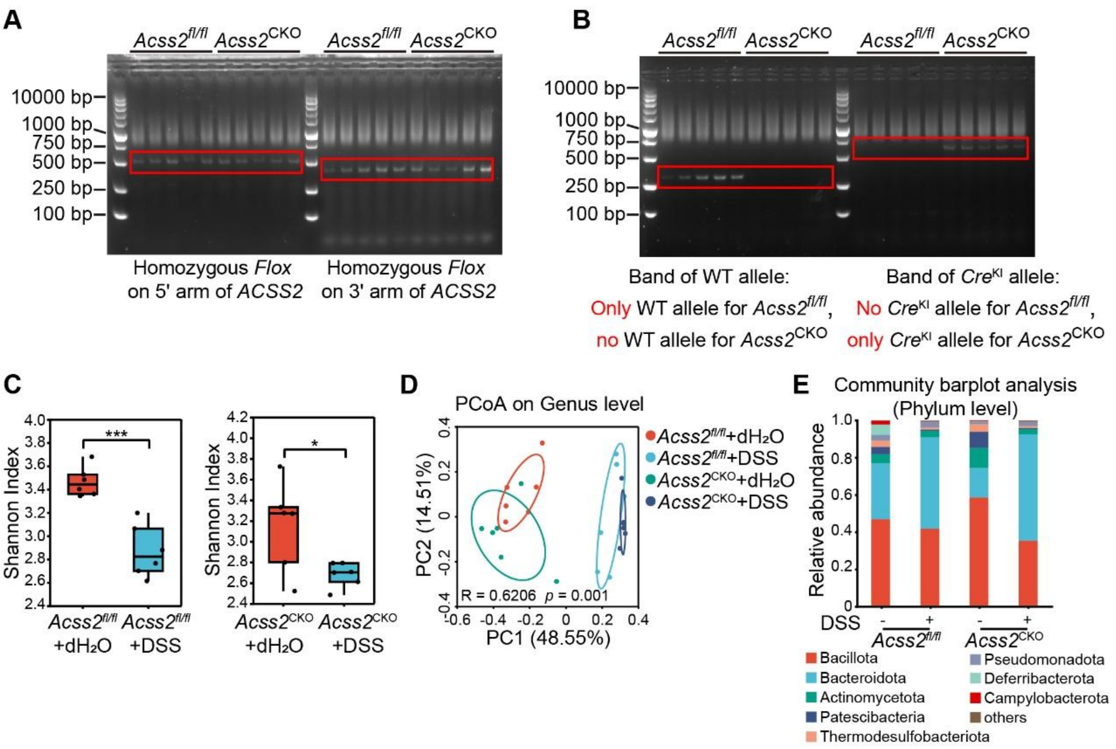

Figure S2. The microbiota composition of *Acss2*$^{fl/fl}$ mice and *Acss2*^{CKO} mice under homeostatic or colitis conditions. A, B) Representative genotyping results for (A) *Flox* and (B) *Cre* expression in *Acss2*$^{fl/fl}$ mice and *Acss2*^{CKO} mice. C-G) The microbiota composition changed significantly in both *Acss2*$^{fl/fl}$ mice and *Acss2*^{CKO} mice with colitis (n = 6 per group). C) The Shannon index of indicated experimental groups. D) The PCoA of indicated experimental groups. E) The microbiota composition analysis in phylum level. Values are mean ± SD. *$p < 0.05$, ***$p < 0.001$, determined by two-tailed Student's *t*-test (C) and principal coordinate analysis (D).

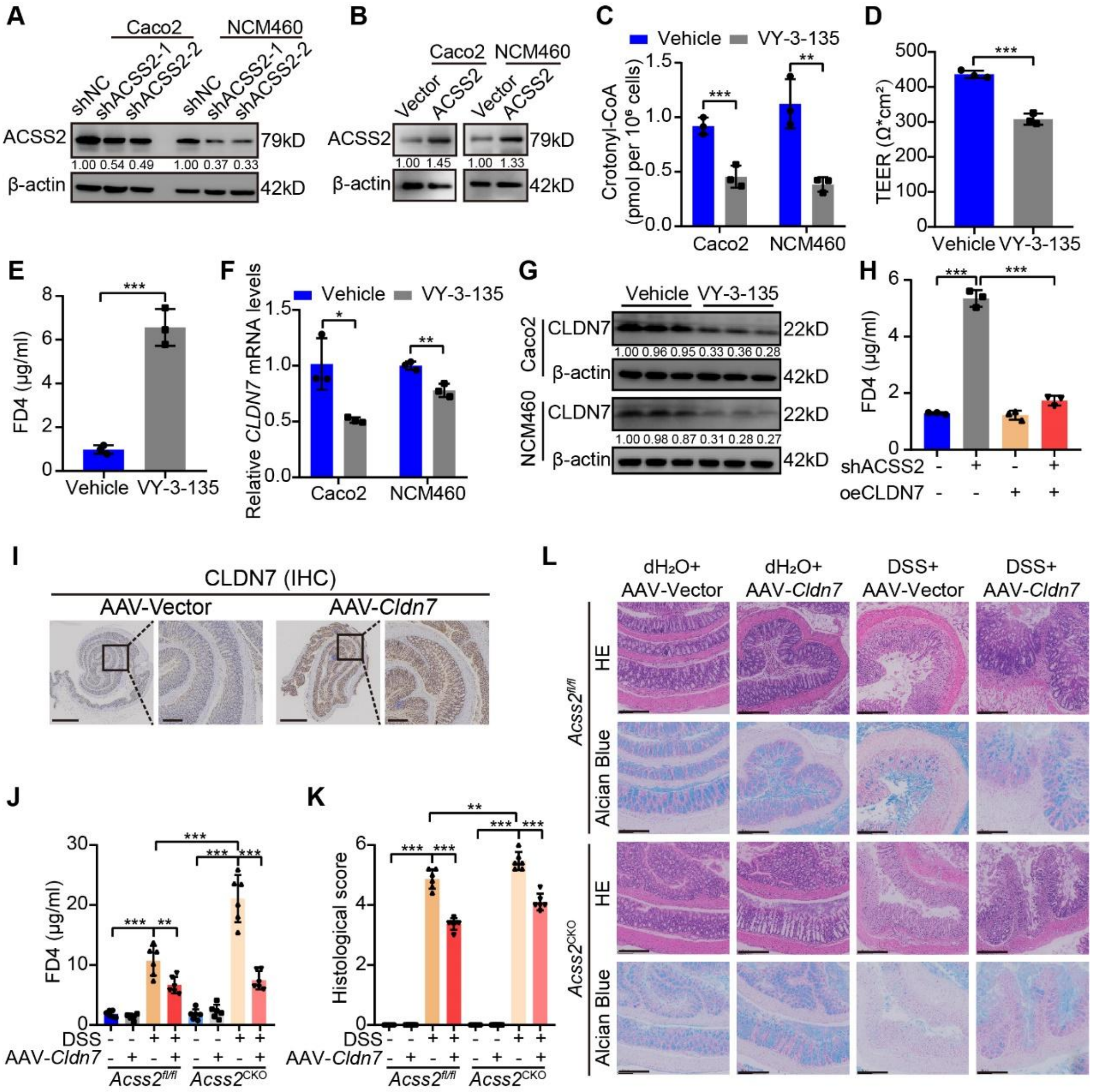

Figure S3. ACSS2 inhibition downregulates CLDN7 expression and impairs intestinal barrier function. A, B) Immunoblotting of ACSS2 expression in control and ACSS2-knockdown or ACSS2-overexpression cells. C) The intracellular concentration of crotonyl-CoA in cells with VY-3-135 (1 μM) treatment. D, E) TEER and FD4 measurement in monolayers of Caco2 cells with VY-3-135 (1 μM) treatment. F, G) Decreased CLDN7 expression in cells with VY-3-135 (1 μM) treatment, as detected by F) qRT-PCR and G) immunoblotting. H) FD4 measurement in monolayers of Caco2 cells with indicated transfection. I) Representative IHC staining images of CLDN7 in colon tissues from mice administrated with AAV9-vector or AAV9-*Cldn7*. Scale bar =

250 μm (left) and 50 μm (right). J) The plasma FD4 level of mice from indicated groups. K, L) Histological score and representative HE/Alcian blue staining images of colon tissues from indicated groups. Scale bar = 100 μm. Relative band intensities are annotated below respective lanes (A, B, G). Values are mean ± SD. *$p < 0.05$, **$p < 0.01$, ***$p < 0.001$, determined by two-tailed Student's *t*-test (C-F) and one-way ANOVA with Bonferroni's post hoc test (H, J, K).

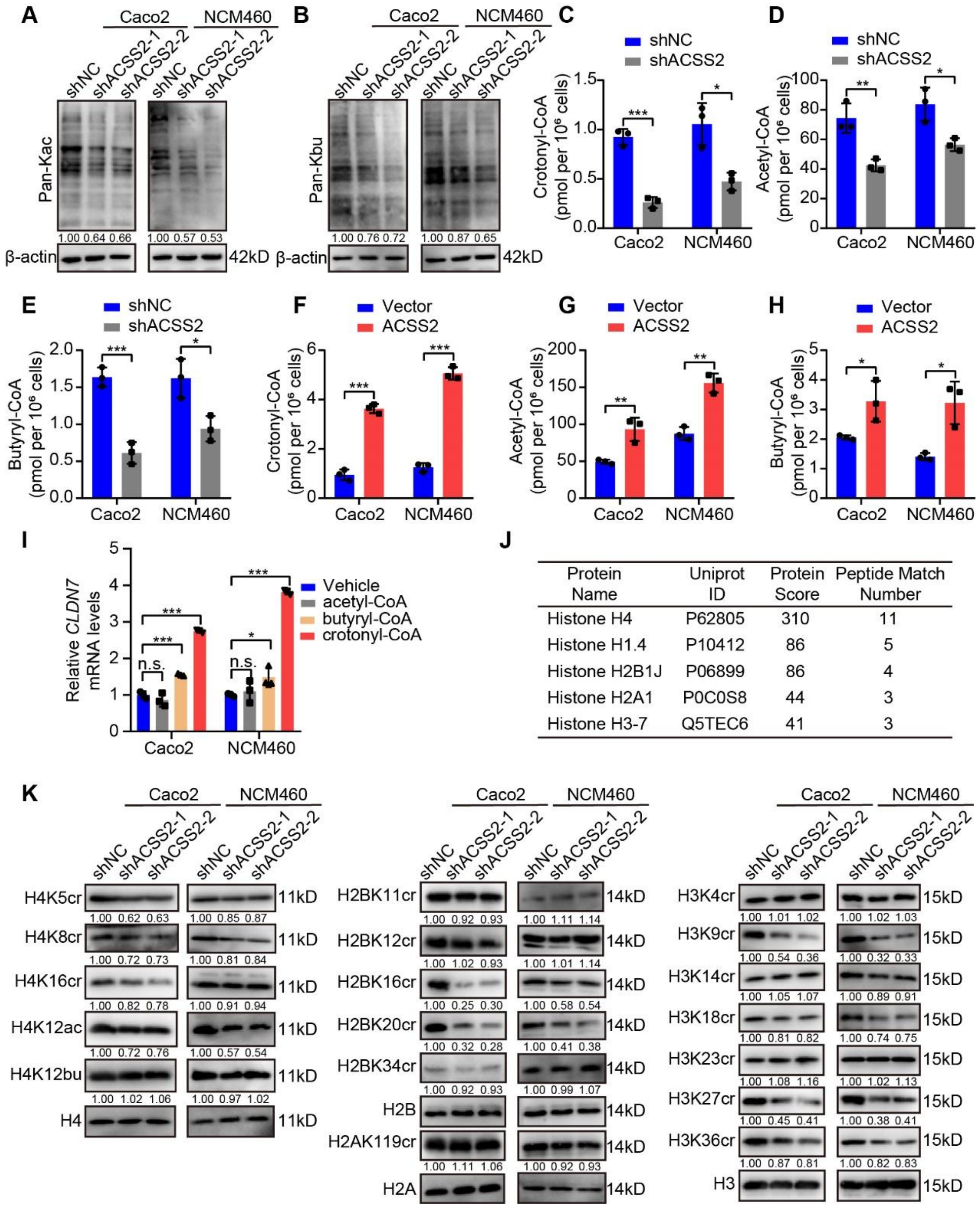

Figure S4. ACSS2 modulates histone crotonylation. A, B) Immunoblotting of Pan-Kac and Pan-Kbu level in control or ACSS2-knockdown cells. C-H) The intracellular concentration of C, F) crotonyl-CoA, D, G) acetyl-CoA, and E, H) butyryl-CoA in cells with ACSS2-knockdown or ACSS2-overexpressed, detected by mass spectrometry. I) Relative mRNA level of *CLDN7* in Caco2 and NCM460 cells treated with indicated acyl-CoA (200 μM) for 48 h. J) The crotonylated histone abundance detected by mass

spectrometry. K) Immunoblotting of different histone modification sites in Caco2 and NCM460 with ACSS2 knockdown. Relative band intensities are annotated below respective lanes (A, B, K). Values are mean ± SD. n.s. (not significant, $p > 0.05$), *$p < 0.05$, **$p < 0.01$, ***$p < 0.001$, determined by two-tailed Student's *t*-test (C-H) and one-way ANOVA with Bonferroni's post hoc test (I).

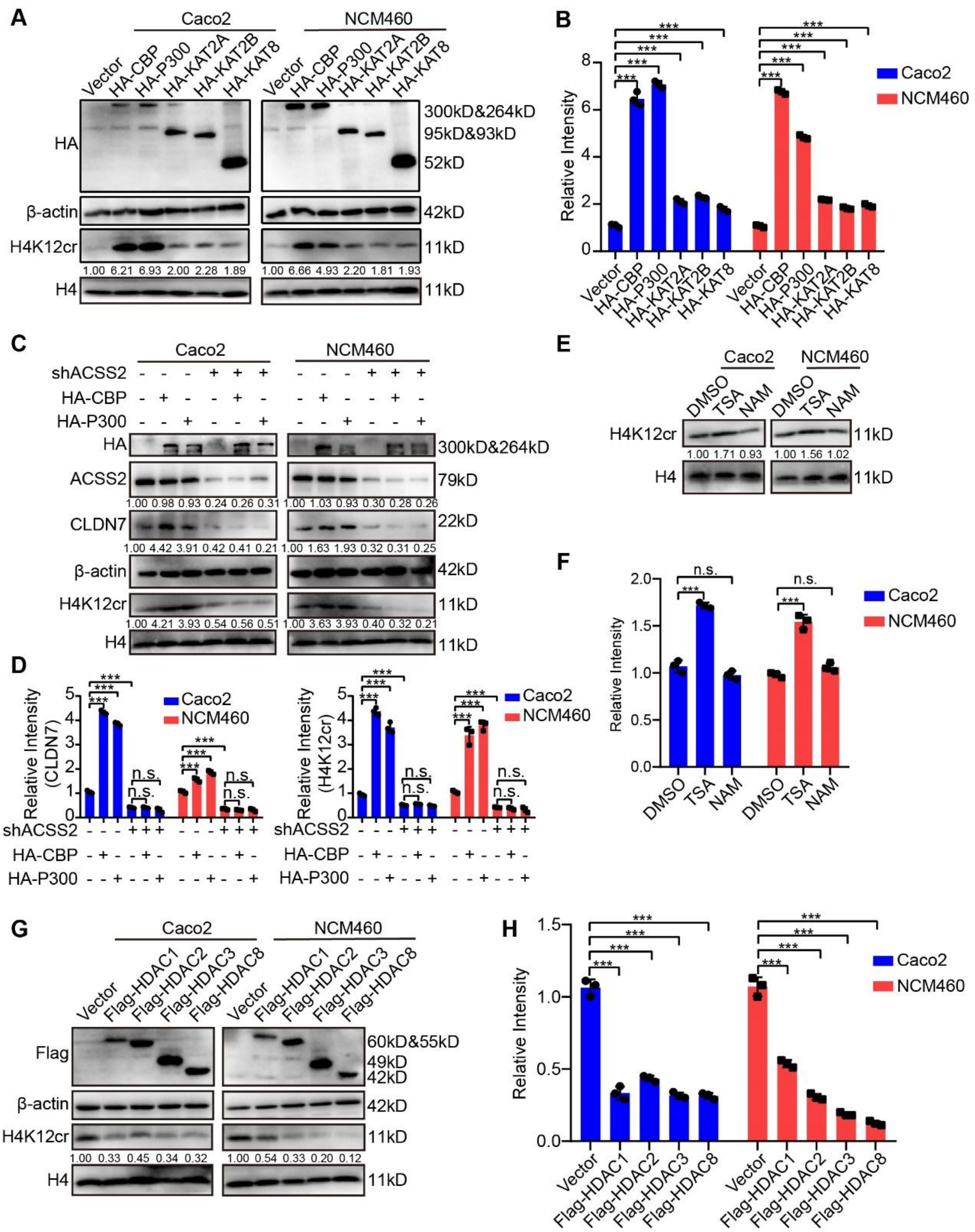

Figure S5. H4K12cr is modulated by several writers and erasers. A, B) Immunoblotting and statistical analysis of H4K12Cr in cells with indicated "writers" transfection. C, D) Immunoblotting and statistical analysis of cells with indicated transfection, probed by specific antibodies. E, F) Immunoblotting and statistical analysis of H4K12cr in cells treated with TSA (1 μM) or NAM (10 mM) for 10 h. G-

H) Immunoblotting and statistical analysis of H4K12Cr in cells with indicated “erasers” transfection. Relative band intensities are annotated below respective lanes. Values are mean ± SD. n.s. (not significant, $p > 0.05$), ***$p < 0.001$, determined by one-way ANOVA with Bonferroni’s post hoc test (B, D, F, H).

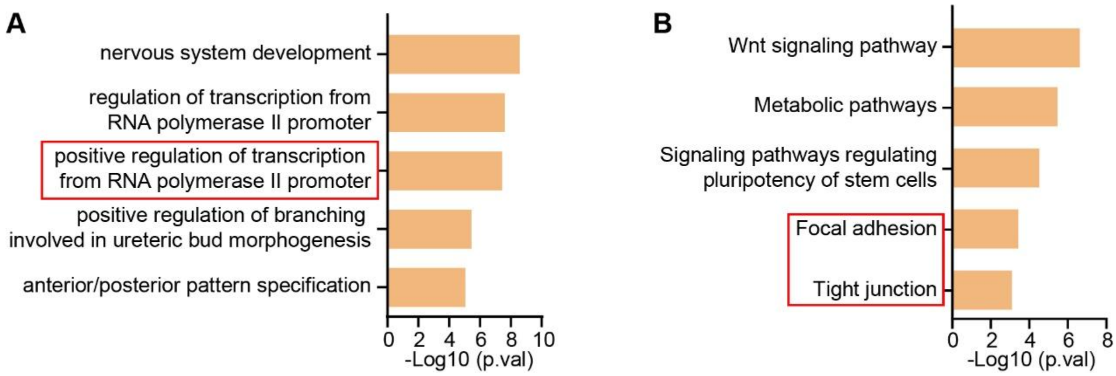

Figure S6. The biological processed and pathways regulated by H4K12cr. A) GO and B) KEGG analysis results of genes with peak loss in NCM460 with ACSS2 knockdown.

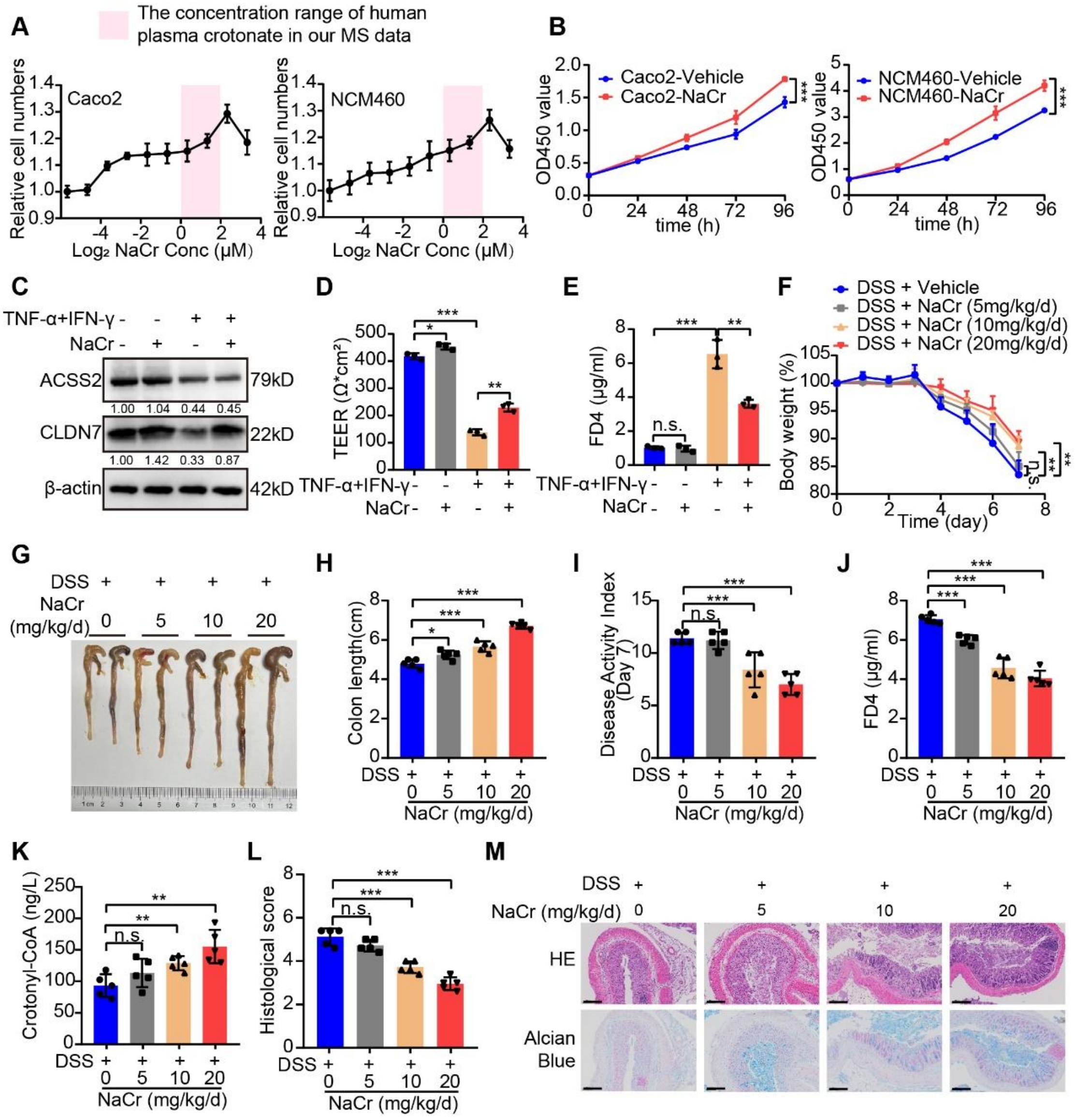

Figure S7. NaCr alleviates colitis in a concentration-dependent manner. A) Cell viability assays of Caco2 and NCM460 cells with gradient concentrations of NaCr treatment. B) Cell proliferation ability of cells treated with NaCr (10 mmol/L), assessed using CCK8 assays. C-E) C) Immunoblotting of indicated proteins, D) TEER, and E) FD4 measurement in monolayers of Caco2 cells treated with TNF-α (10 ng/ml), IFN-γ (10 ng/ml) and NaCr (10 mmol/L). Relative band intensities are annotated below respective lanes. F-M) NaCr alleviated colitis in a concentration-dependent manner (n = 5 per group). F) The mice body weight curves from indicated experimental groups.

G, H) Representative colon images and the colon length statistics from each group. I) The DAI statistics for mice from indicated groups. J) The plasma FD4 level in mice from indicated experimental groups. K) The plasma crotonyl-CoA concentration in mice from indicated experimental groups. L-M) Histological score and representative HE/Alcian blue staining images of colon tissues from indicated groups. Scale bar = 100 μm. Values are mean ± SD. n.s. (not significant, $p > 0.05$), $^{*}p < 0.05$, $^{**}p < 0.01$, $^{***}p < 0.001$, determined by repeated measures ANOVA with Bonferroni's post hoc test (B, F) and one-way ANOVA with Bonferroni's post hoc test (D, E, H-L).

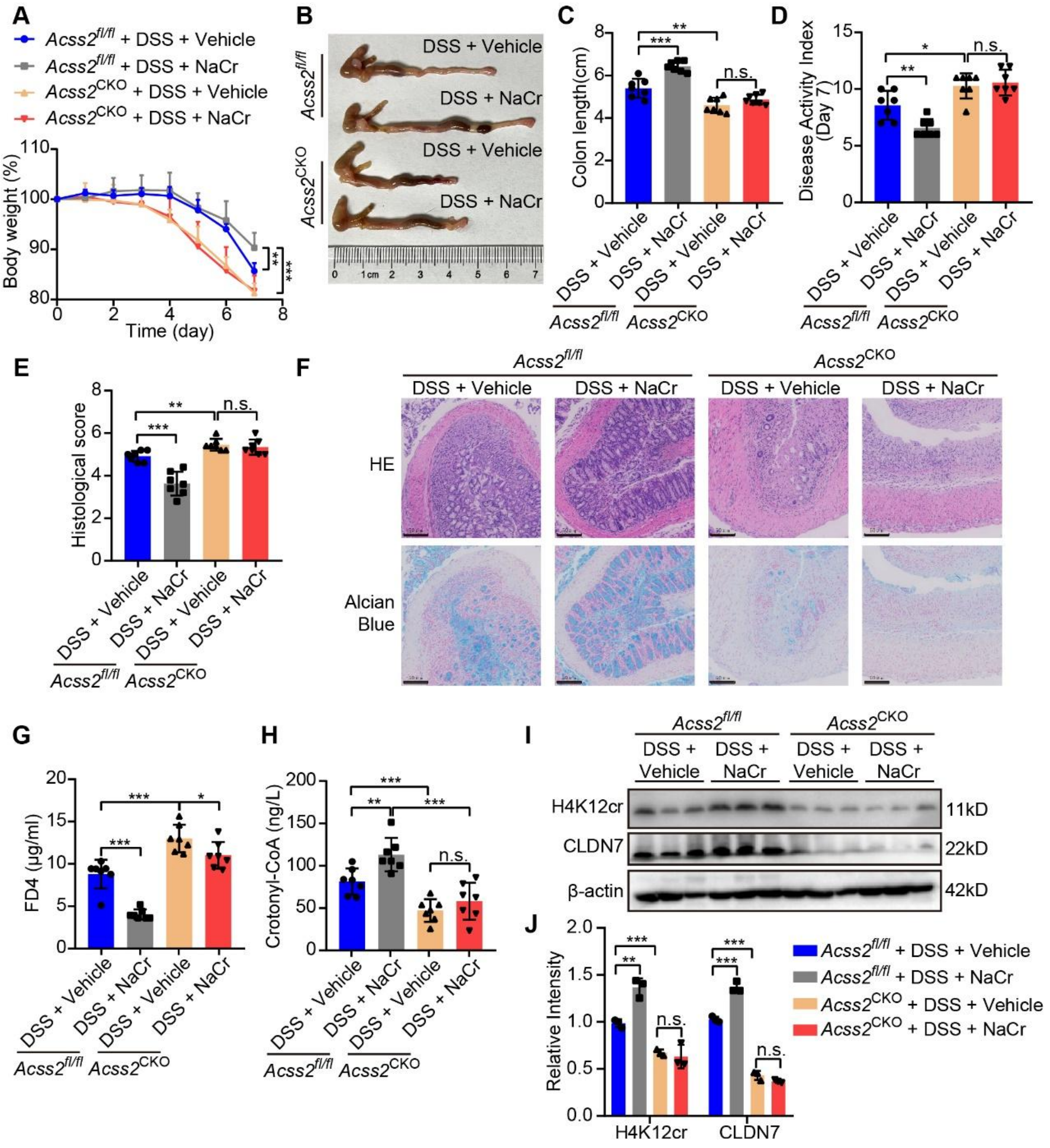

Figure S8. Sodium crotonate alleviates colitis dependent on the enzymatic activity of ACSS2. A) The mice body weight curves from indicated experimental groups. B, C) Representative colon images and colon length statistics from each group. D) The DAI statistics for mice from indicated groups. E, F) Histological score and representative HE/Alcian blue staining images of colon tissues from indicated groups. Scale bar = 50 μm. G) The plasma FD4 level in indicated groups. H) The plasma crotonyl-CoA concentration in mice from indicated experimental groups. I, J) Immunoblotting and

statistical analysis of H4K12cr and CLDN7 in colon tissues from indicated groups. The concentration of NaCr used *in vivo* were 20 mg/kg/d (n = 7 per group). Values are mean ± SD. *$p < 0.05$, **$p < 0.01$, ***$p < 0.001$, determined by repeated measures ANOVA with Bonferroni's post hoc test (A), one-way ANOVA with Bonferroni's post hoc test (C-E, G, H, J).

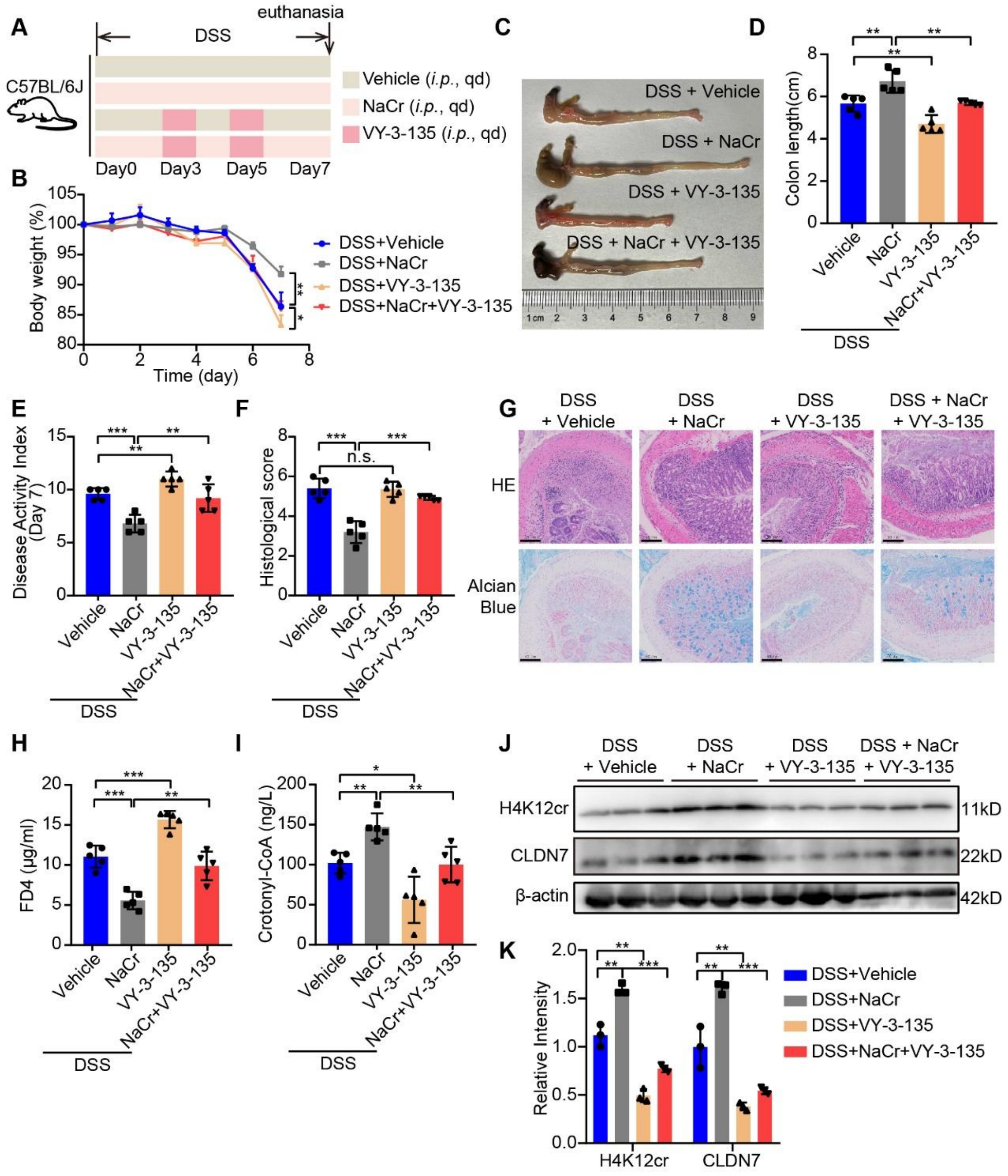

Figure S9. NaCr alleviates colitis dependent on the enzymatic activity of ACSS2. A) The administrative strategy of VY-3-135 and NaCr (n = 8 for each group). B) The mice body weight curves from indicated experimental groups. C, D) Representative colon images and colon length statistics from each group. E) The DAI statistics for mice from indicated groups. F, G) Histological score and representative HE/Alcian blue staining images of colon tissues from indicated groups. Scale bar = 50 μm. H) The

plasma FD4 level in mice from indicated experimental groups. I) The plasma crotonyl-CoA concentration in mice from indicated experimental groups. J, K) Immunoblotting and statistical analysis of H4K12Cr and CLDN7 in colon tissues from indicated groups. Values are mean ± SD. n.s. (not significant, $p > 0.05$), *$p < 0.05$, **$p < 0.01$, ***$p < 0.001$, determined by repeated measures ANOVA with Bonferroni's post hoc test (B) and one-way ANOVA with Bonferroni's post hoc test (D-F, H, I, K).

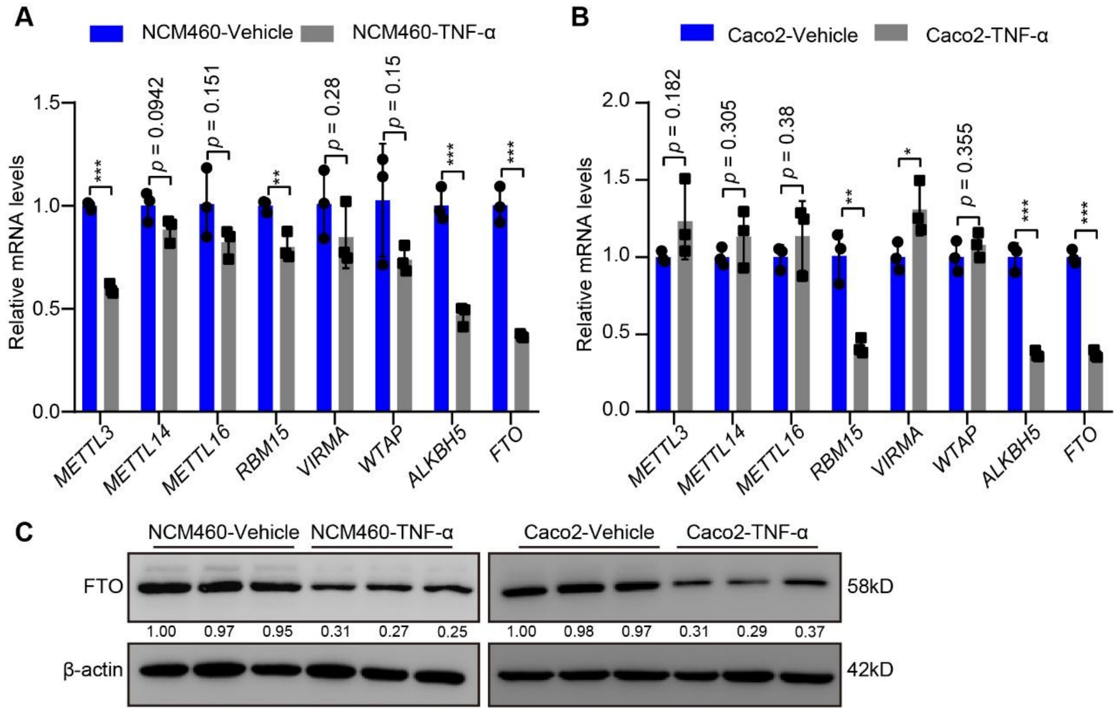

Figure S10. TNF-α reduces FTO expression level. A, B) Relative mRNA levels of enzymes involved in m6A modification in Caco2 and NCM460 treated with TNF-α (50 ng/mL). C) Immunoblotting of FTO in Caco2 and NCM460 with TNF-α treatment (50 ng/mL). Relative band intensities are annotated below respective lanes. Values are mean ± SD. **p* < 0.05, ***p* < 0.01, ****p* < 0.001, determined by two-tailed Student's *t*-test (A, B).

Supplementary Tables

Table S1. The clinicopathologic characteristics of IBD patients and healthy control for IHC staining.

Characteristics	CD patients (n = 24)	UC patients (n = 9)	Healthy colon from CRC patients (n = 25)
Gender			
Male, n (%)	17 (70.8)	5 (55.6)	17 (68)
Female, n (%)	7 (29.2)	4 (44.4)	8 (32)
Age at sample collection (years), median (range)	34 (18-57)	50 (21-58)	37 (18-60)
Biopsy Location, n (%)			
Terminal ileum	17 (70.8)	0 (0)	/
Ascending colon	7 (29.2)	0 (0)	16 (64)
Transverse colon	0 (0)	1 (11.1)	0 (0)
Descending colon	0 (0)	3 (33.3)	4 (16)
Sigmoid	0 (0)	0 (0)	0 (0)
Rectum	0 (0)	5 (55.6)	5 (20)
Medications used in the past six months, n (%)			
TNF-α inhibitor	5 (20.8)	4 (44.4)	/
5-aminosalicylic acid	11 (45.8)	6 (66.7)	/
Azathioprine	4 (16.7)	6 (66.7)	/
Steroids	4 (16.7)	5 (55.6)	/
Antibiotic	6 (25)	2 (22.2)	/
CDAI, median (range)	72 (30-280)	/	/
Disease Location, n (%)			
L1, ileal	0 (0)	/	/
L2, colonic	17 (70.8)	/	/
L3, ileocolonic	7 (29.2)	/	/
Disease Behavior, n (%)			
B1, Non-stricturing, non-penetrating	6 (25)	/	/
B2, Stricturing	2 (8.3)	/	/
B3, Penetrating	16 (66.7)	/	/
Perianal Disease, n (%)	5 (20.8)	/	/
Mayo score, median (range)	/	5 (3-12)	/

Montreal Classification for UC			
E1, proctitis	/	0 (0)	/
E2, left-sided colitis	/	0 (0)	/
E3, extensive colitis	/	9 (100)	/
Severity for UC (Truelove and Witts's criteria)			
Mild	/	1 (11.1)	/
Moderate	/	2 (22.2)	/
Severe	/	6 (66.7)	/

Table S2. The clinicopathologic characteristics of CD patients and healthy control for qRT-PCR.

Characteristics	CD patients (n = 26)	Healthy colon from CRC patients (n = 26)
Gender		
Male, n (%)	16 (61.5)	14 (53.8)
Female, n (%)	10 (38.5)	12 (46.2)
Age at sample collection (years), median (range)	26 (14-70)	65 (25-84)
Biopsy Location, n (%)		
Terminal ileum	13 (50)	/
Ascending colon	8 (30.8)	6 (23.1)
Transverse colon	1 (3.8)	1 (3.8)
Descending colon	4 (15.4)	2 (7.7)
Sigmoid	0 (0)	7 (26.9)
Rectum	0 (0)	10 (38.5)
Medications used in the past six months, n (%)		
TNF-α inhibitor	10 (38.5)	/
5-aminosalicylic acid	15 (57.7)	/
Azathioprine	5 (19.2)	/
Steroids	5 (19.2)	/
Antibiotic	9 (34.6)	/
CDAI, median (range)	85.5 (32-258)	/
Disease Location, n (%)		
L1, ileal	4 (15.4)	/
L2, colonic	0 (0)	/
L3, ileocolonic	22 (84.6)	/
Disease Behavior, n (%)		
B1, Non-stricturing, non-penetrating	12 (46.2)	/
B2, Stricturing	9 (34.6)	/
B3, Penetrating	5 (19.2)	/
Perianal Disease, n (%)	12 (46.2)	/

Table S3. The clinicopathologic characteristics of CD patients and healthy control for plasma collection.

Characteristics	CD patients (n = 28)	Healthy volunteers (n = 10)
Gender		
Male, n (%)	20 (71.4)	6 (60)
Female, n (%)	8 (28.6)	4 (40)
Age at sample collection (years), median (range)	26 (18-42)	26 (24-29)
CDAI, median (range)	79 (40-200)	/
Medications used in the past six months, n (%)		
TNF-α inhibitor	12 (42.9)	/
5-aminosalicylic acid	13 (46.4)	/
Azathioprine	8 (28.6)	/
Steroids	5 (17.9)	/
Antibiotic	15 (53.6)	/
Disease Location, n (%)		
L1, ileal	0 (0)	/
L2, colonic	2 (7.1)	/
L3, ileocolonic	26 (92.9)	/
Disease Behavior, n (%)		
B1, Non-stricturing, non-penetrating	17 (60.7)	/
B2, Stricturing	5 (17.9)	/
B3, Penetrating	6 (21.4)	/
Perianal Disease, n (%)	10 (35.7)	/

Table S4. The primers used for qRT-PCR.

Species	Gene	Forward primer	Reverse primer
Mouse	*Acads*	TTGCCGAGAAGGAGTTGGTC	AGGTAATCCAAGCCTGCACC
	Acox1	GCCATTCGATACAGTGCTGTGAG	CCGAGAAAGTGGAAGGCATAGG
	Echs1	ATCACCCGGGTCAAGAAACC	TCGCCAGCATAGATGATATCACA
	Acss2	CCATTGCCACACCAGACTAC	TCAGCCACCGTAGATGTATCC
	Gcdh	AAGAACCCGCTTGCAAAACC	ACCATAACCTGGTGCTTGCG
	Acox3	GACGCATCTCCATCATCAGCAT	GCCAGAGCATGGATCTCACGT
	β-actin	CATTGCTGACAGGATGCAGAAGG	TGCTGGAAGGTGGACAGTGAGG
Human	*ACSS2*	CAAGTGTGTCAGTTCAGCAATG	CCACAAGCTCTGGGATCATAGG
	CLDN1	CCCTATGACCCCAGTCAATG	ACCTCCCAGAAGGCAGAGA
	CLDN2	CACCGCTAGGATGTAGCCCACAAGT	AAACACTTGTGGGCTACATCCTAGC
	CLDN3	CGCGAGAAGAAGTACACGG	CCTTAGACGTAGTCCTTGCGG
	CLDN4	CGCATCAGGACTGGCTTTATCTC	CAGCGCGATGCCCATTA
	CLDN5	CTGACCTTCTCCTGCCACTAG	GAAGCGAAATCCTCAGTCTGACA
	CLDN7	AAAGTGAAGAAGGCCCGTATA	TAATGTTGGTAGGGATCAAAGG
	CLDN8	GGCTGTTTCTTGGTGGTGTT	CACGCAATTCATCCACAGTC
	CLDN12	TGCGAACAAGAAGTTTGAGC	GGTGGATGGGAGTACAATGG
	CLDN15	CCATCTTCGAGAACCTCTGG	CCTGAATATACCCAGAGAGGG
	CLDN18	TTCCATCCCAGTACCAAAGC	CCGTTCTTTCCCCAGACATA
	OCLN	ACTTCAGGCAGCCTCGTTAC	CCTGATCCAGTCCTCCTCCA
	TJP1	GGAGTCTGCCATTACACGGT	AGGTCTCTGCTGGCTTGTTT
	METTL3	TTGTCTCCAACCTTCCGTAGT	CCAGATCAGAGAGGTGGTGTAG
	METTL14	AGTGCCGACAGCATTGGTG	GGAGCAGAGGTATCATAGGAAGC
	METTL16	TTCTGTCAAGGTCGGACAATG	CAGCACCACGAATGTTATGGG
	RBM15	GTGAGGACTCGACTTCCCG	GCCGCTATCGGTCTTTCCG
	VIRMA	TACTTTGAGCCCATTTCTCCTGA	GGAATACTGTCTACTGTTCGTCG
	WTAP	CTTCCCAAGAAGGTTCGATTGA	TCAGACTCTCTTAGGCCAGTTAC
	ALKBH5	CGGCGAAGGCTACACTTACG	CCACCAGCTTTTGGATCACCA
	FTO	ACTTGGCTCCCTTATCTGACC	TGTGCAGTGTGAGAAAGGCTT
	β-actin	CACCATTGGCAATGAGCGGTTC	AGGTCTTTGCGGATGTCCACGT

Table S5. The antibodies used for immunoblotting and immunohistochemistry.

Antibodies	Source	Identifier	Usage
Pan-Kcr	PTMBIO	#PTM-501	WB (1:1000); IHC (1:200)
Pan-Kac	PTMBIO	#PTM-105RM	WB (1:1000)
Pan-Kbu	PTMBIO	#PTM-301RM	WB (1:1000)
ACSS2	Cell Signaling Technology	#3658	WB (1:1000); IHC (1:200)
CLDN7	Invitrogen	#34-9100	WB (1:1000); IHC (1:100)
β-actin	Servicebio	#GB15003	WB (1:3000)
H4K5cr	PTMBIO	#PTM-521RM	WB (1:1000)
H4K8cr	PTMBIO	#PTM-522RM	WB (1:1000)
H4K12cr	PTMBIO	#PTM-530	WB (1:1000)
H4K16cr	PTMBIO	#PTM-532	WB (1:1000)
H4K12ac	Proteintech	#83095-1-RR	WB (1:1000)
H4K12bu	PTMBIO	#PTM-314	WB (1:1000)
H4	Proteintech	#16047-1-AP	WB (1:1000)
H2BK11cr	PTMBIO	#PTM-529	WB (1:1000)
H2BK12cr	PTMBIO	#PTM-528	WB (1:1000)
H2BK16cr	PTMBIO	#PTM-533	WB (1:1000)
H2BK20cr	PTMBIO	#PTM-534	WB (1:1000)
H2BK34cr	PTMBIO	#PTM-514	WB (1:1000)
H2B	Proteintech	#27740-1-AP	WB (1:1000)
H2AK119cr	PTMBIO	#PTM-543	WB (1:1000)
H2A	Proteintech	#39346	WB (1:1000)
H3K4cr	PTMBIO	#PTM-527	WB (1:1000)
H3K9cr	PTMBIO	#PTM-539	WB (1:1000)
H3K14cr	PTMBIO	#PTM-537	WB (1:1000)
H3K18cr	PTMBIO	#PTM-540	WB (1:1000)
H3K23cr	PTMBIO	#PTM-519	WB (1:1000)
H3K36cr	PTMBIO	#PTM-536RM	WB (1:1000)
H3	Proteintech	#17168-1-AP	WB (1:1000)
HA	Cell Signaling Technology	#3724S	WB (1:1000)
Flag	Cell Signaling Technology	#14793S	WB (1:1000)
FTO	Abcam	#ab126605	WB (1:1000)

Table S6. The primers used for mice genotyping.

Mice	Primer No.	Sequence
Acss2$^{fl/fl}$	T016449-F1	TCACTTGAGAACTTCCTACCTTAGCC
	T016449-R1	AGACACTGTGCCCGCTCAACATAT
	T016449-F2	ATTCAAAGCTTAGAAGCCTTGGTAGG
	T016449-R2	GGAAGCAGAATGAGCTGTTAGTGAAAC
Vil1-Cre	T004714-F1A	TCTACTGGAGGAGGACAAACTGGTC
	T004714-R1A	TATCTCCTTCCACAGGCATCTTCCA
	T004714-F1	GGGCAGTCTGGTACTTCCAAGCT
	T004714-R1	ATATCCCCTTGTTCCCTTTCTGC

Table S7. The reagents used for cell treatment.

Reagents	Source	Identifier
VY-3-135	MedChemExpress (Shanghai, China)	#HY-145953
Trichostatin A (TSA)	MedChemExpress (Shanghai, China)	#HY-15144
Nicotinamide (NAM)	MedChemExpress (Shanghai, China)	#HY-B0150
TNF-α	PeproTech (Suzhou, China)	#300-01A
IFN-γ	PeproTech (Suzhou, China)	#300-02
IL-1β	PeproTech (Suzhou, China)	#200-01B
IL-6	PeproTech (Suzhou, China)	#200-06
IL-8	PeproTech (Suzhou, China)	#200-08M
IL-11	PeproTech (Suzhou, China)	#200-11
IL-17	PeproTech (Suzhou, China)	#200-17
acetyl-CoA	Sigma-Aldrich (St. Louis, Mo, USA)	#A2056
butyryl-CoA	Sigma-Aldrich (St. Louis, Mo, USA)	#B1508
crotonyl-CoA	Sigma-Aldrich (St. Louis, Mo, USA)	#28007
crotonic acid	Sigma-Aldrich (St. Louis, Mo, USA)	#113018
Actinomycin D	MedChemExpress (Shanghai, China)	#HY-17559

Table S8. The primers used for shRNA construction.

Species	Gene	Forward primer	Reverse primer
Human	ACSS2-sh1	CCGGGCTTCTGTTCTGGGTCTGAATCTCGAGATTCAGACCCAGAACAGAAGCTTTTTG	AATTCAAAAAGCTTCTGTTCTGGGTCTGAATCTCGAGATTCAGACCCAGAACAGAAGC
	ACSS2-sh2	CCGGCGGTTCTGCTACTTTCCCATTCTCGAGAATGGGAAAGTAGCAGAACCGTTTTTG	AATTCAAAAACGGTTCTGCTACTTTCCCATTCTCGAGAATGGGAAAGTAGCAGAACCG
	FTO-sh	CCGGTCACGAATTGCCCGAACATTACTCGAGTAATGTTCGGGCAATTCGTGATTTTTG	AATTCAAAAATCACGAATTGCCCGAACATTACTCGAGTAATGTTCGGGCAATTCGTGA

Table S9. The plasmids used for gene overexpression.

Plasmids	Source	Identifier
pCMV3-C-HA-ACSS2	Sino Biological (Beijing, China)	#HG22964-CY
pLV3-CMV-CREBBP (human)-3×HA-Puro	MiaoLingBio (Wuhan, China)	#P58813
pSG5-HA-p300	MiaoLingBio (Wuhan, China)	#P41347
pLV3-CMV-3×HA-KAT2A (human)-CopGFP-Puro	MiaoLingBio (Wuhan, China)	#P50506
pLV3-CMV-KAT2B (human)-3×HA-Puro	MiaoLingBio (Wuhan, China)	#P60203
pCMV-KAT8 (human)-3×HA-Neo	MiaoLingBio (Wuhan, China)	#P63393
pEnCMV-HDAC1 (human)-3×FLAG-SV40-Neo	MiaoLingBio (Wuhan, China)	#P32870
pCMV-HDAC2 (human)-3×FLAG-Neo	MiaoLingBio (Wuhan, China)	#P6907
pCMV-HDAC3 (human)-FLAG-SV40-Neo	MiaoLingBio (Wuhan, China)	#P38134
pCMV-HDAC8 (human)-FLAG-SV40-Neo	MiaoLingBio (Wuhan, China)	#P37989
pLV3-CMV-FTO (human)-3×FLAG-Puro	MiaoLingBio (Wuhan, China)	#P43834

165 **Table S10. The endogenous proteins with lysine crotonylation modification.**

No.	Protein Name	*Uniprot* ID	prot_ score	prot_ matches	No.	Protein Name	*Uniprot* ID	prot_ score	prot_ matches
1	ODP2	P10515	1165	50	40	H2B1J	P06899	86	4
2	K2C1	P04264	1027	29	41	GRP75	P38646	82	3
3	ODPX	O00330	827	31	42	G3PT	O14556	81	5
4	K1C10	P13645	520	16	43	STON2	Q8WXE9	79	3
5	K1C9	P35527	487	11	44	DCD	P81605	76	1
6	K1C19	P08727	473	17	45	ENOA	P06733	74	1
7	ODPB	P11177	338	15	46	RL7	P18124	72	2
8	K22E	P35908	315	16	47	PRDX1	Q06830	67	3
9	H4	P62805	310	11	48	NUCL	P19338	61	2
10	EP300	Q09472	283	10	49	K1C27	Q7Z3Y8	60	2
11	K2C8	P05787	283	16	50	KAT7	O95251	56	3
12	K2C6A	P02538	269	10	51	IF2B1	Q9NZI8	55	1
13	TBA1A	Q71U36	223	8	52	CPSM	P31327	54	2
14	K1C18	P05783	213	8	53	LDHA	P00338	54	1
15	ODPA	P08559	210	13	54	GCSP	P23378	52	2
16	ACTB	P60709	206	12	55	PGAM2	P15259	50	2
17	DLDH	P09622	203	8	56	AKT2	P31751	50	3
18	K2C5	P13647	181	8	57	CLPX	O76031	49	1
19	ODB2	P11182	174	12	58	ADRO	P22570	49	1
20	ALBU	P02768	169	6	59	HBD	P02042	46	1
21	TBB5	P07437	162	4	60	ROCK2	O75116	45	2
22	TBB4B	P68371	148	3	61	ING4	Q9UNL4	45	1
23	HSP7C	P11142	147	5	62	SAHH	P23526	45	2
24	G3P	P04406	146	6	63	H2A1	P0C0S8	44	3
25	JADE3	Q92613	139	5	64	ASSY	P00966	43	1
26	K1C14	P02533	136	7	65	PIWL4	Q7Z3Z4	43	1
27	BIP	P11021	135	3	66	NNTM	Q13423	42	3
28	DPYL2	Q16555	127	5	67	ATPB	P06576	42	2
29	K1C16	P08779	127	6	68	H3-7	Q5TEC6	41	3
30	IGG1	P0DOX5	123	18	69	DYH1	Q9P2D7	40	4
31	CBP	Q92793	122	6	70	RS9	P46781	40	1
32	CH60	P10809	109	4	71	RYR3	Q15413	39	2
33	LAP2A	P42166	105	4	72	IMB1	Q14974	39	1
34	RL19	P84098	92	2	73	GEMI5	Q8TEQ6	39	1
35	ATPA	P25705	90	6	74	RL11	P62913	38	1
36	PDIA6	Q15084	87	1	75	KHDR2	Q5VWX1	38	2
37	NINL	Q9Y2I6	87	2	76	RL13	P26373	38	3
38	RS3	P23396	87	2	77	NPFF2	Q9Y5X5	38	2
39	H1.4	P10412	86	5	78	KAT6B	Q8WYB5	38	5
79	FERM1	Q9BQL6	37	2	122	WDFY3	Q8IZQ1	31	1

80	ROAA	Q99729	37	1	123	KV37	A0A075B6H7	31	1
81	EMD	P50402	37	1	124	PCDC2	Q9Y5I4	31	1
82	PRDX6	P30041	37	1	125	CDC6	Q99741	31	1
83	OXND1	Q96HP4	37	2	126	PKP3	Q9Y446	31	1
84	GRIK5	Q16478	36	2	127	LAMC2	Q13753	31	1
85	IL6RA	P08887	36	2	128	SZT2	Q5T011	31	1
86	HMMR	O75330	36	1	129	SNW1	Q13573	30	1
87	ARL3	P36405	36	1	130	SENP3	Q9H4L4	30	2
88	SBNO1	A3KN83	36	2	131	ODAD2	Q5T2S8	30	1
89	EF1A3	Q5VTE0	36	2	132	HTRA1	Q92743	30	1
90	SOGA1	O94964	36	2	133	BL1S4	Q9NUP1	30	1
91	FXL13	Q8NEE6	35	3	134	KBTBB	O94819	30	2
92	ODO2	P36957	35	1	135	ZSCA4	Q8NAM6	29	1
93	MYO1H	Q8N1T3	35	2	136	PSME4	Q14997	29	4
94	GAR1A	Q6NXP2	35	2	137	RLA0	P05388	29	2
95	DIRA2	Q96HU8	35	1	138	NLRC5	Q86WI3	29	1
96	ACOHC	P21399	35	1	139	TRHDE	Q9UKU6	29	1
97	URGCP	Q8TCY9	34	2	140	SH3K1	Q96B97	29	1
98	HOATZ	Q6PI97	34	1	141	RL4	P36578	29	4
99	WASC4	Q2M389	34	3	142	KI20A	O95235	29	2
100	LASP1	Q14847	34	1	143	CWC25	Q9NXE8	29	2
101	PUR2	P22102	34	1	144	TITIN	Q8WZ42	29	9
102	GDAS1	Q5U4N7	34	1	145	TATD2	Q93075	29	2
103	PRP6	O94906	33	2	146	PPE2	O14830	29	1
104	RS27A	P62979	33	1	147	SPTN4	Q9H254	28	1
105	TMC1	Q8TDI8	33	1	148	BCORL	Q5H9F3	28	1
106	SCN5A	Q14524	33	1	149	RL10A	P62906	28	1
107	CC146	Q8IYE0	33	2	150	KLC2	Q9H0B6	28	1
108	PIDD1	Q9HB75	33	2	151	DSRAD	P55265	28	2
109	ANM7	Q9NVM4	33	1	152	DYH6	Q9C0G6	28	1
110	RHBT1	O94844	33	1	153	GIMA6	Q6P9H5	28	1
111	RO52	P19474	33	1	154	PHB2	Q99623	28	1
112	MYH16	Q9H6N6	33	1	155	CE112	Q8N8E3	28	2
113	GNPTA	Q3T906	33	1	156	CAPG	P40121	28	1
114	RS19	P39019	32	2	157	KV127	A0A075B6S5	28	1
115	APOB	P04114	32	2	158	EF2	P13639	28	1
116	ZN468	Q5VIY5	32	1	159	SYNJ2	O15056	28	1
117	DDX24	Q9GZR7	32	3	160	TNR21	O75509	28	2
118	ZBT7B	O15156	32	1	161	ZN559	Q9BR84	27	1
119	TSYL1	Q9H0U9	32	2	162	IQCE	Q6IPM2	27	2
120	NAV1	Q8NEY1	32	2	163	LPH	P09848	27	1
121	SMG1	Q96Q15	31	3	164	KCNA7	Q96RP8	27	2
165	YETS2	Q9ULM3	27	1	208	PGK1	P00558	24	1
166	RS13	P62277	27	1	209	RS4X	P62701	24	2

167	AFAM	P43652	27	1	210	R13P3	Q6NVV1	24	1
168	PUS1	Q9Y606	27	2	211	GABT	P80404	24	1
169	DEN4C	Q5VZ89	27	2	212	NCOA1	Q15788	24	2
170	DAPLE	Q9P219	27	1	213	WDR11	Q9BZH6	24	2
171	YTDC1	Q96MU7	27	1	214	XRCC5	P13010	24	2
172	MYH7	P12883	27	1	215	CYFP1	Q7L576	24	1
173	S2539	Q9BZJ4	27	1	216	AG10A	Q5BKT4	24	1
174	KVD24	A0A075B6R9	27	2	217	ATG12	O94817	24	2
175	N4BP2	Q86UW6	27	2	218	CC020	Q8ND61	23	1
176	JAK1	P23458	26	1	219	PRAX	Q9BXM0	23	3
177	RS7	P62081	26	1	220	SYCP2	Q9BX26	23	2
178	KRT85	P78386	26	1	221	SYHM	P49590	23	1
179	TRI25	Q14258	26	1	222	KS6A4	O75676	23	2
180	HEP2	P05546	26	1	223	ARHG8	Q7Z628	23	1
181	NECA2	Q7Z6G3	26	1	224	UBF1	P17480	23	1
182	OD3L1	Q8IXM7	26	1	225	TTPA	P49638	23	1
183	AATC	P17174	26	1	226	IF3M	Q9H2K0	23	1
184	PAR16	Q8N5Y8	26	1	227	SATT	P43007	23	2
185	SYNM	Q96I59	25	1	228	CHSTA	O43529	23	3
186	ALLC	Q8N6M5	25	1	229	GBF1	Q92538	23	1
187	DRC11	Q86XH1	25	2	230	STN1	Q9H668	23	1
188	RL3	P39023	25	1	231	NDUC1	O43677	23	1
189	MCM6	Q14566	25	2	232	ZN827	Q17R98	23	1
190	RNBP6	O60518	25	1	233	NUD17	P0C025	22	1
191	MITD1	Q8WV92	25	1	234	WDR36	Q8NI36	22	1
192	RHG21	Q5T5U3	25	2	235	CRERF	Q8IUR6	22	1
193	CCD63	Q8NA47	25	2	236	CE295	Q9C0D2	22	1
194	KCIP4	Q6PIL6	25	2	237	LR10B	A6NIK2	22	2
195	BBIP1	A8MTZ0	25	1	238	NUD13	Q86X67	22	1
196	HD	P42858	25	1	239	PLK1	P53350	22	2
197	PP1RA	Q96QC0	25	1	240	BEX5	Q5H9J7	21	1
198	O52R1	Q8NGF1	25	1	241	AMPE	Q07075	21	1
199	HPS6	Q86YV9	25	1	242	CNTRL	Q7Z7A1	21	3
200	TCAF1	Q9Y4C2	25	1	243	ZDHC5	Q9C0B5	21	1
201	RB6I2	Q8IUD2	25	1	244	SIK3	Q9Y2K2	21	1
202	TLL1	O43897	25	1	245	PRR34	Q9NV39	21	1
203	KEAP1	Q14145	25	3	246	AGRA2	Q96PE1	21	1
204	ODBA	P12694	24	1	247	MUC18	P43121	21	3
205	ASPM	Q8IZT6	24	4	248	FOLC	Q05932	21	1
206	VIPR2	P41587	24	1	249	GOGA2	Q08379	20	2
207	RXRA	P19793	24	1	250	ABCA1	O95477	20	1
251	MORC1	Q86VD1	20	3					
252	EXT1	Q16394	20	1					
253	SPTN5	Q9NRC6	20	3					

254	ADCK5	Q3MIX3	20	3
255	THOC2	Q8NI27	19	2
256	VP13A	Q96RL7	19	2
257	STAR9	Q9P2P6	19	2
258	CA167	Q5SNV9	19	5
259	SEPT2	Q15019	19	1
260	LONM	P36776	19	2
261	KCNG2	Q9UJ96	18	2
262	UD17	Q9HAW7	18	1
263	POK6	Q9BXR3	18	3
264	IGDC4	Q8TDY8	18	1
265	PWWP4	A0A494C071	17	1
266	IGS22	Q8N9C0	17	1
267	GP176	Q14439	17	2
268	CNTLN	Q9NXG0	16	1
269	OBSCN	Q5VST9	16	3
270	GFPT1	Q06210	16	2
271	COQ6	Q9Y2Z9	15	1
272	ANKH	Q9HCJ1	15	1
273	BAP29	Q9UHQ4	15	1
274	ERLN1	O75477	15	1
275	RM11	Q9Y3B7	14	2
276	PREX1	Q8TCU6	14	3
277	B3AT	P02730	14	1

166
